# Single‐cell RNA‐sequencing technology demonstrates the heterogeneity between aged prostate peripheral and transitional zone

**DOI:** 10.1002/ctm2.1084

**Published:** 2022-10-17

**Authors:** Qiuxia Yan, Miao Wang, Haoran Xia, Cao Dai, Tongxiang Diao, Yingfei Wang, Huimin Hou, Hong Zhang, Ming Liu, Xingbo Long

**Affiliations:** ^1^ Peking University Fifth School of Clinical Medicine Beijing China; ^2^ Department of Urology Beijing Hospital National Center of Gerontology Beijing China; ^3^ Department of General Surgery The Third Affiliated Hospital Sun Yat‐sen University Guangzhou Guangdong China; ^4^ Department of Urology Shandong Provincial Hospital Affiliated to Shandong First Medical University Jinan China; ^5^ Beijing Hongying Primary School Beijing China; ^6^ Institute of Cardiovascular Sciences and Key Laboratory of Molecular Cardiovascular Sciences Peking University Health Science Center Beijing China; ^7^ Department of Urology Sun Yat‐sen University Cancer Center Guangzhou Guangdong China; ^8^ State Key Laboratory of Oncology in South China Collaborative Innovation Center for Cancer Medicine Sun Yat‐Sen University Cancer Center Guangzhou Guangdong China

**Keywords:** aged prostate, benign prostatic hyperplasia, heterogeneity, NOTCH signaling, prostate cancer, single‐cell RNA‐sequencing

## Abstract

**Background:**

Identifying cellular and functional heterogeneity within aged prostate is critical for understanding the spatial distribution of prostate diseases.

**Methods:**

Aged human prostate peripheral zone (PZ) and transitional zone (TZ) tissues were used for single‐cell RNA‐sequencing. Results were validated by immunofluorescence staining.

**Results:**

We found that club/hillock epithelial cells, compared with other epithelial cells, had significantly higher NOTCH signaling activity and expressed higher levels of neuro‐stems but lower androgen‐related genes. These cells were primarily found in the TZ and provided a stem‐like niche around the proximal prostate ducts. Significant heterogeneity was observed in the aged luminal population. A novel TFF3+ luminal subtype with elevated MYC and E2F pathway activities was observed, primarily in the PZ. Further analysis revealed that epithelial cells in the TZ had higher levels of stem‐ and inflammation‐related pathway activities but lower androgen/lineage‐related pathway activities than those in the PZ. Notably, the activation of MYC, E2F and DNA repair pathways significantly increased in PZ luminal cells. In the immune landscape, we found that the immune microenvironment in the TZ is more complex and disordered with more infiltration of NK and Treg cells. CD8 T cell and macrophage in the TZ exhibit both inflammation activation and suppression phenotypes. In the stroma, the TZ had a higher fibroblast density, and fibroblasts in the TZ exhibited stronger transcriptome activity in immunity and proliferation. Ligand–receptor interaction analysis revealed that fibroblasts could contribute to a NOTCH signaling niche for club/hillock cells in the TZ and balance the prostate immune microenvironment. The activation of stem properties, inflammatory infiltration and loss of androgen/lineage activity are prominent features distinguishing the TZ from PZ.

**Conclusions:**

Our study explains the heterogeneity between the TZ and PZ of aged prostate, which may help understand the spatial distribution of prostate diseases and establish a foundation for novel target discovery.

## Graphical abstract

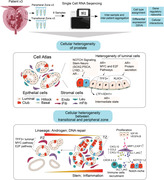

**Abbreviations**
BPH: benign prostatic hyperplasia
CCA: canonical correlation analysis
CNV: copy number variation
DAPI: 4′,6‐diamidino‐2‐phenylindole, and dilactate
ECM: extracellular matrix
GSVA: gene set variation analysis
HE: hematoxylin‐eosin
MDSCs: yeloid‐derived suppressor cells
PCa: prostate cancer
PZ: peripheral zone
scRNA‐seq: single‐cell RNA sequencing
tSNE: distributed stochastic neighbour embedding
TZ: transitional zone

## 1 BACKGROUND

The prostate is a heterogeneous gland that harbours phenotypically and functionally diverse cell subpopulations. The histological composition of the human prostate primarily includes the epithelium and fibre‐muscular stroma,^1^ comprising anatomically characteristic zone‐based differences. McNeal anatomically divided the prostate into three glandular regions: the peripheral zone (PZ), the central zone and the transition zone (TZ).^2^ Previous research has revealed significant zone‐based differences in the incidence and development of prostate diseases.^3^ Benign prostatic hyperplasia (BPH) and prostate cancer (PCa) are two prostate diseases commonly observed in elderly patients. These diseases primarily affect men aged 70 years.^4^, ^5^ BPH primarily occurs in the TZ, whereas PZ has been described more frequently in terms of cancer development.^6^ Although this clinical phenomenon has long been recognised, the underlying reasons and mechanisms have not yet been elucidated. Many studies have attempted to determine the morphological and molecular differences between TZ and PZ.^7^, ^8^ However, efforts to investigate these variances have primarily focused on anatomical zones rather than cellular composition and functional heterogeneity.

Traditionally, the prostate is an exocrine gland in which prostatic epithelia are composed of three major cell types: secretory luminal, basal and rare neuroendocrine cells.^9^ Multipotent basal cells may give rise to all three prostate epithelial cell lineages.^10^, ^11^ Luminal cells are considered to be functional secretory epithelial.^12^ In addition to these cell types, various rare stem cells have been detected in benign and malignant prostate tissues.^13^, ^14^ These findings suggest that the heterogeneity of the prostate is greater than previously understood. Notably, using single‐cell RNA sequencing (scRNA‐seq) technology to examine normal prostate tissues from young donors, Henry et al. determined the molecular classification and molecular markers of diverse types of prostate cells, revealing two new types of epithelial cells, namely, ‘club’ and ‘hillock’. ^15^

However, the subjects used by Henry et al.^15^ were young, and the prostate drastically changes with age, commonly causing disease in older patients. Therefore, further identification of cellular and functional heterogeneity within an aged prostate is critical for understanding the spatial distribution, development and progression of prostate diseases, which may help to identify novel therapeutic targets.

## 2 METHODS

### 2.1 Samples for scRNA‐sequencing

All patient‐derived tissues were collected in compliance with the rules and regulations of the Ethics Committee of Beijing Hospital (2019BJYYEC‐226‐02), and informed consent was obtained from all patients.

Three paired hormonally intact benign prostate TZ and PZ tissues were obtained from patients who underwent radical prostatectomy for early‐stage PCa (T2a). The patients included in the study were all Asian with a mean age of 65.6. These patients did not exhibit any PCa or BPH symptoms. PCa was diagnosed based on an elevated PSA‐level during physical screening. Preoperative MRI indicated that all PCa patients had single lesions. They were also free of preoperative treatment such as 5‐alpha‐reductase inhibitors, androgen deprivation therapy or other medical therapy. The demographic and clinical information of each patient is shown in Figure S1.

When collecting the tissues, the prostate gland was transversely sectioned at 0.5‐cm intervals from the apex to the base. The postoperative gross sample of the whole prostate confirmed that all three patients had a single lesion, and the maximum cross‐sectional area of the lesion is less than 8.3 mm^2^ (Figure S1). Two lesions were located in the PZ and one lesion was located in the TZ (Figure S1). Several benign‐like regions of the TZ and PZ in the contralateral lobe where the tumour was located in the tumour‐free layer were sampled and digested into a single‐cell suspension to minimise the effect of tumour tissue. The section layer and location at which the samples were collected were recorded. After tissue sampling, each prostate section was fixed and stained with hematoxylin‐eosin (HE) to further confirm the location and approximate size of the tumour lesion (Figure S1). The histomorphology of the selected regions was evaluated by a pathologist based on histological analysis, HE and AMACR staining (Figures S1 and S2A). Only tissues confirmed by HE without AMACR expression (PCa marker), prostate intraepithelial neoplasia, chronic and acute inflammatory lesions and BPH nodules with glandular tissue, the corresponding single‐cell suspensions were qualified for single‐cell sequencing (Figures S1 and S2A). Regarding the study by Nevoux et al.,^16^ we described the lesion location and sampling area (Figure S1). The mean distance between the lesion and the TZ and PZ sampling area was 28.8 and 31.4 mm, respectively (Figure S1). The tumour and sampling location of each patient are shown in Figure S1.

### 2.2 Prostate isolation and enzymatic digestion

Prostates were isolated as previously described.^15^, ^17^ In brief, prostates were harvested and subsequently digested with collagenase type II (2 mg/mL, Life Technologies, cat:17101‐015) supplemented with 10 μmol rock inhibitor (Y27632, Abcam; cat: ab120129), 1 nmol Dihydrotestosterone (Cerilliant; cat: D‐073) and 2 unit DNase I (Invitrogen; cat:18068‐015) in Hank's Balanced Salt Solution (Gibco; cat:24020‐117) for 2 h at 37°C. The cells were digested again in TrypLE (Life Technologies, cat:12605‐010) with 10 μmol rock inhibitor (Y27632) at 37°C for 10 min until a single‐cell suspension was obtained. The cells were resuspended in RBC lysis buffer (BioLegend; cat:420301) for 3 min on ice. Finally, the cells were washed twice with 0.02% PBS‐BSA and assessed for viability using trypan blue (Gibco; cat:15250061) staining. All experiments were performed with a minimum of 80% viable cells.

### 2.3 Immunohistochemistry

Fluorescence immunohistochemistry was performed as described previously.^18^ Briefly, 5 μm paraffin sections were deparaffinised in xylene and hydrated through a series of ethanol washes. Heat‐mediated antigen retrieval was performed by boiling the slides in 10 mmol sodium citrate (pH 6.0) for 20 min in a conventional microwave oven. The tissues were washed with a solution containing 25 mmol Tris‐HCl (pH 7.5), 140 mmol NaCl, 2.7 mmol KCl and 0.1% Tween‐20 (TBSTw), and nonspecific binding sites were blocked for 1 h in TBSTw containing 1% blocking reagent (Roche Diagnostics, Indianapolis, IN), 5% normal goat serum and 1% bovine serum albumin fraction 5 (RGBTw). The tissues were incubated overnight at 4°C with primary antibodies diluted in RGBTw, washed several times with TBSTw and incubated with secondary antibodies diluted in RGBTw for 1 h at room temperature. Following several washes with TBSTw, the tissue sections were incubated with 4′,6‐diamidino‐2‐phenylindole, and dilactate (DAPI) to visualise cell nuclei and mounted in phosphate‐buffered saline containing 80% glycerol and 0.2% n‐propyl gallate. Images were obtained using a Keyence BZ‐X700 microscope (Keyence, Osaka, Japan). For antibody information, see Table S1 in the Supporting Information.

### 2.4 Single‐cell sequencing

In our study, two scRNA‐seq platforms (10× Genomics and Singleron) were employed to verify the stability of our scRNA data and cross‐platform validation. (1) Four samples were subjected to droplet‐based scRNA‐seq. The Chromium Single‐cell 3′‐Library, Gel Bead & Multiplex Kit and Chip Kit (10× Genomics, Pleasanton, CA, USA) were used to construct a chromium single‐cell 3′‐library, according to the manufacturer's instructions. Cell suspensions were loaded onto a chromium single‐cell chip along with reverse transcription master mix and single‐cell 3′‐gel beads to obtain 2000–8000 single cells per reaction. Samples were processed using a 10× Genomics V2 barcoding chemistry kit. Following cell lysis, first‐strand cDNA synthesis and amplification were performed according to the manufacturer's instructions, with a cDNA amplification set for 12 cycles. The libraries were sequenced using the Illumina HiSeq X Ten system. (2) Two samples were subjected to Singleron scRNA‐seq. Single‐cell suspensions at a concentration of 1 × 10^5^ cells/mL in PBS (HyClone) were prepared and loaded onto microfluidic devices. ScRNA‐seq libraries were constructed according to the Singleron GEXSCOPE™ protocol using the GEXSCOPE™ Single‐Cell RNA Library Kit (Singleron Biotechnologies).^19^ Individual libraries were diluted to 4 nmol and pooled for sequencing using the Illumina HiSeq X system.

Single‐cell sequencing mapping strategies of two platforms are shown in the Materials and Methods in the Supporting Information.

Table S2 provides a summary of the basic sequencing and mapping information. A median of 595 919 962 reads per sample had a sequencing saturation of 78.6%, with an average of 47 457 reads and 1315 genes per cell.

### 2.5 Statistical analysis

All statistical analyses and graph generation were performed using R (version 3.6.1).

Details regarding bioinformatic processes and evaluation of immunostaining are described in the Materials and Methods in the Supporting Information.

## 3 RESULTS

### 3.1 Single‐cell sequencing and cell‐type identification

Three paired, hormonally intact, histopathologically confirmed, benign prostate TZ PZ tissues were collected and digested into single cells. Before single‐cell sequencing analysis, we calculated the proliferation index of the TZ and PZ by Ki67 and PCNA staining. The results show both Ki67 and PCNA positive cells were not high in the TZ and PZ and no significant statistical difference can be observed between them (Figure S2B).

**FIGURE 1:**
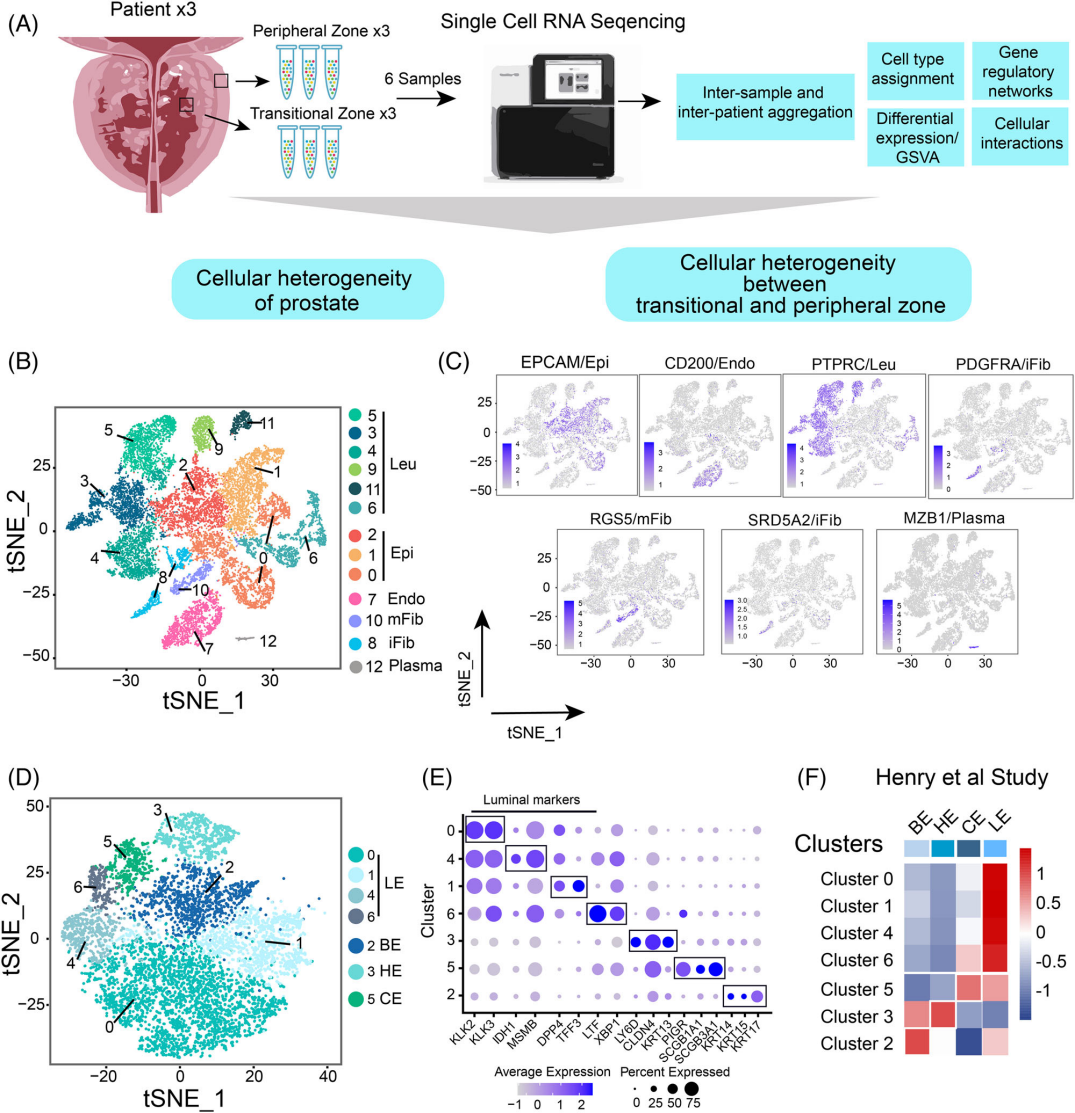
Single‐cell gene expression profiling of the prostate. (A) Workflow and design of our study. (B) t‐SNE plot of aggregated single‐cell RNA sequencing data from six prostate specimens. Cells were divided into 13 clusters (indicated by colours). (C) Cells were annotated based on known lineage‐specific marker genes such as epithelial cells (Epi), endothelial cells (Endo), leukocytes (Leu), myofibres (mFib) and inflammatory fibroblasts (iFib). (D) t‐SNE plot of epithelial cells. Epithelial cells were further divided into seven clusters (indicated by colours). LE: luminal epithelial. HE: hillock epithelial. CE: club epithelial. BE: basal epithelial. E: Dot plot of marker genes for each epithelial clusters in our scRNA‐Seq data. E: SingleR analysis of the correlation of each epithelial cluster of previously published young donor prostate epithelial cell types^15^

Figure 1A shows the study design. After performing quality control and removing stress and doublet cells (Figures S3A and B), 18 315 qualified single cells were obtained. By using canonical correlation analysis (CCA), we integrated all these cells from different samples and platforms (see Materials and Methods in the Supporting Information). Figures S3C–E show the cell sample, anatomy and platform origin. The results showed that the cell composition and distribution were relatively stable, and only weak batch effects were observed across samples and platforms after integration.

Subsequently, 18 315 qualified single cells were grouped into 13 major clusters in a t‐distributed stochastic neighbour embedding (tSNE) plot (Figure 1B) with an average of 3425 transcripts and 1307 genes per cluster (Figure S3F).

Cluster‐specific genes were used to annotate cell types as epithelial (EPCAM), endothelial (CD200) and two types of fibroblasts [inflammatory fibroblasts (iFib: PDGFRA) and myofibroblasts (mFib: RGS5)], leukocytes (PTPRC) and plasma cells (MZB1) (Figure 1C). Notably, SRD5A2, the target of BPH therapy, was expressed exclusively in the iFib group (Figure 1C). To verify the stability of CCA integration, the Harmony method (see Materials and Methods in the Supporting Information) was also used to integrate the samples, and the results showed that the two integration methods obtained similar cell cluster results (Figures S4A and B).

EPCAM+ epithelial cells were grouped into seven sub‐clusters (Figure 1D), for which Figure 1E shows marker genes. Sub‐clusters 2, 3 and 5 were annotated as basal (KRT 14), hillock (KRT 13) and club (SCGB3A1) cells, respectively, based on marker genes and correlations with previously published data^15^ (Figures 1E and F). Sub‐clusters 0, 1, 4 and 6 were annotated as luminal clusters. All highly expressed luminal markers (KLK3) were correlated with a previously published luminal epithelial phenotype^15^ (Figure 1E).

Leukocytes were divided and annotated as T cells (CD3D), B cells (MS4A1), neutrophils (S100A9) and macrophages (CD68) (Figures S4C–E); T cells were further annotated as CD4 T (CD4), CD8 T (CD8), Treg (FOXP3), NK T (CD8; GNLY) and NK cells (GNLY) (Figures S4F–H).

We used single‐cell DNA copy number variation (CNV) profiles to ensure that our analysis was restricted to normal tissue.^20^ We integrated our epithelial cells with previously published young‐donor prostate^15^ and PCa^21^ epithelial cells. The young‐donor prostate^15^ and PCa^21^ epithelial cells were used as normal and malignant references, respectively. Results indicated that our data had a CNV profile and CNV score like those of the young‐donor prostate and was significantly different from the PCa CNV profile (Figures S5A and B). In addition, the expression of several PCa‐specific genes was notably low in our and young‐donor prostate data, which was significantly lower than those in malignant luminal cells (Figure S5C). This finding indicated that the samples did not contain tumour cells.

Finally, we integrated our aged prostate scRNA‐seq data with young‐donor prostate scRNA‐seq data.^15^ The results showed that the major cell types in the young‐donor prostate gland corresponded to those in the aged prostate gland (Figure S6A). However, great difference could be observed in the proportion of cell types between the young‐donor and aged prostate (Figure S6B). Compared to the young‐donor prostate, a higher proportion of luminal epithelial cells and leukocyte cells but a lower proportion of basal cells were observed in the aged prostate (Figure S6B). The above data shows that there are differences in cellular proportion between young‐donor and aged prostates; therefore, it is necessary to further explore the heterogeneity of ageing prostate.

### 3.2 Estimated functional heterogeneity and spatial distribution of four prostate epithelial cell types

**FIGURE 2:**
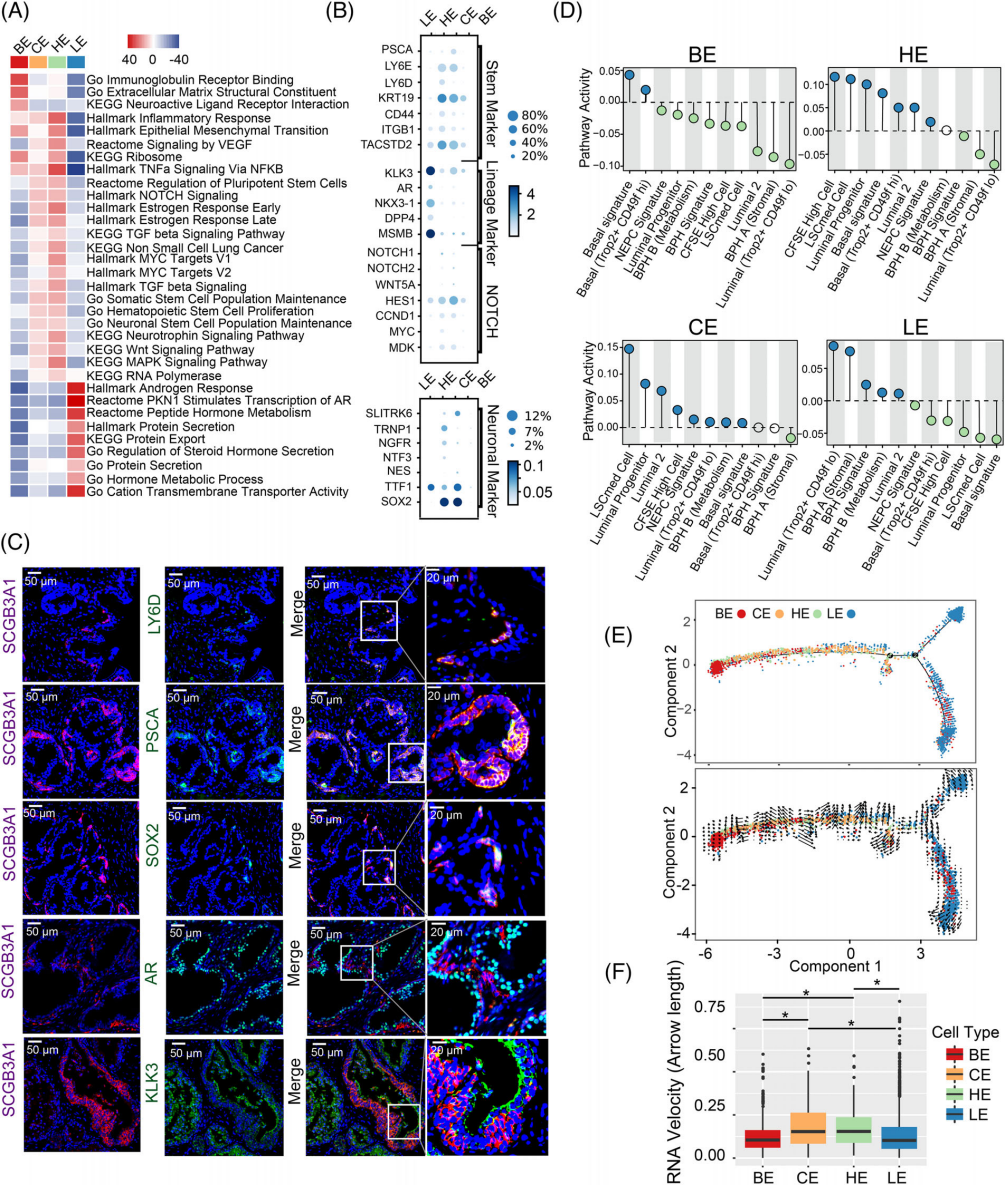
Estimated functional heterogeneity and spatial distribution of four prostate epithelial cells. (A) Heatmap shows differences in pathway activities scored by GSVA per cell between different epithelial cells. (B) Dot plot of gene expression levels in each epithelial population for selected genes (prostate stem, lineage, NOTCH signaling and neuronal markers). (C) Immunofluorescent labelling of prostate stem marker (PSCA, LY6D and SOX2), AR, KLK3 and club (SCGB3A1) cells in prostate transitional zone tissues (magnification: 200×; bar: 50 μm; magnification: 800×; bar: 20 μm). (D) Correlation of epithelial populations to the transcriptomic signature of previously published bulk sequencing data. NEPC Signature (neuroendocrine PCa stem cells^22^); LSCmed Cells (castration‐resistant PCa progenitor cells^23^); CFSE high cells (normal prostate stem cells^14^); and Luminal 2 (mouse luminal prostate stem cells^24^); BPH A and B, and BPH Signature (BPH‐related genes^25^); Basal Trop2+ CD49f hi (basal stem cell signature^26^); Luminal Trop2+ CD49f lo (luminal cell signature^26^); Basal signature.^27^ (E) Upper: Pseudo‐time analysis of epithelial cell state transition in a two‐dimensional state space using highly variable genes inferred by Monocle (version 2). Each dot corresponds to a single cell, as coloured according to its cluster label. Down: Transcriptional activity was estimated by measuring the ratio between unspliced and spliced mRNAs. This so‐called RNA velocity is represented by high‐dimensional vectors; the longer the arrow in the plot is, the higher the transcriptional activity is. (F) Boxplot showing the RNA velocity in the four epithelial cell groups

Through gene set variation analysis (GSVA), we observed that four epithelial cell types have distinct transcriptomes (Figure 2A). Basal cells are enriched in extracellular matrix (ECM) functions. Luminal cells serve as functional secretory epithelial cells and are highly correlated with androgen‐, protein‐secretion‐, and steroid‐related pathways. Notably, the androgen pathway was not highly activated in hillock and club epithelial cells but enriched in the stem, neural stem, NOTCH and oestrogen pathways (Figure 2A). Moreover, the dot plot shows that compared to luminal and basal cells, hillock and club cells express higher levels of prostate stem (e.g. PSCA and LY6D) and neuronal (e.g. SOX2) genes, whereas lineage genes (e.g. AR, KLK3 and NKX3‐1) were expressed at higher levels in luminal cells (Figure 2B). Immunofluorescence further confirmed the co‐expressed of stem neuro‐markers, namely PSCA, LY6D and SOX2, whereas majority of AR and KLK3 were not co‐expressed in KRT13 (hillock)‐ and SCGB3A1 (club)‐strongly positive cells (Figure 2C; Figure S7A).

To further identify the features of the four epithelial clusters, we correlated them with previously published transcriptomes^14^, ^22^, ^23^, ^24^, ^25^, ^26^, ^27^ using QuSAGE GSEA (Figure 2D), and QuSAGE GSEA results were further confirmed by pseudo‐bulk GSEA (Figure S7B). Club and hillock cells are highly correlated with castration‐resistant PCa progenitor cells (LSCmed cells),^23^ normal prostate stem cells (CFSE‐high cells)^14^ and mouse luminal stem cells (Luminal 2)^24^ and are marginally correlated with neuroendocrine PCa stem cells (NEPC signature).^22^ BPH‐related signatures^25^ were enriched in luminal cells (Figure 2D). Next, we applied Monocle2 to sort cells in a linear order. In the plot, club and hillock cells are located between basal and luminal cells (Figure 2E Upper). Notably, we observed high numbers of unspliced RNAs in both club and hillock cells (Figure 2E, Lower, and Figure 2F).

We further analysed the distribution of the four types of epithelial cells. Using immunofluorescence assays, previously published prostate scRNA‐seq data^24^, ^28^ and our prostate scRNA‐seq data, we observed that club and hillock cells were more abundant in the TZ than in the PZ (Figures S8A and S8B). Moreover, the total proportion of the total number of these two cell types increased after androgen deprivation therapy (Figure S8A), and the proportion of club cells was higher in the BPH samples than in the TZ tissues (Figure S8A).

### 3.3 Estimated functional heterogeneity among prostate luminal cells

**FIGURE 3:**
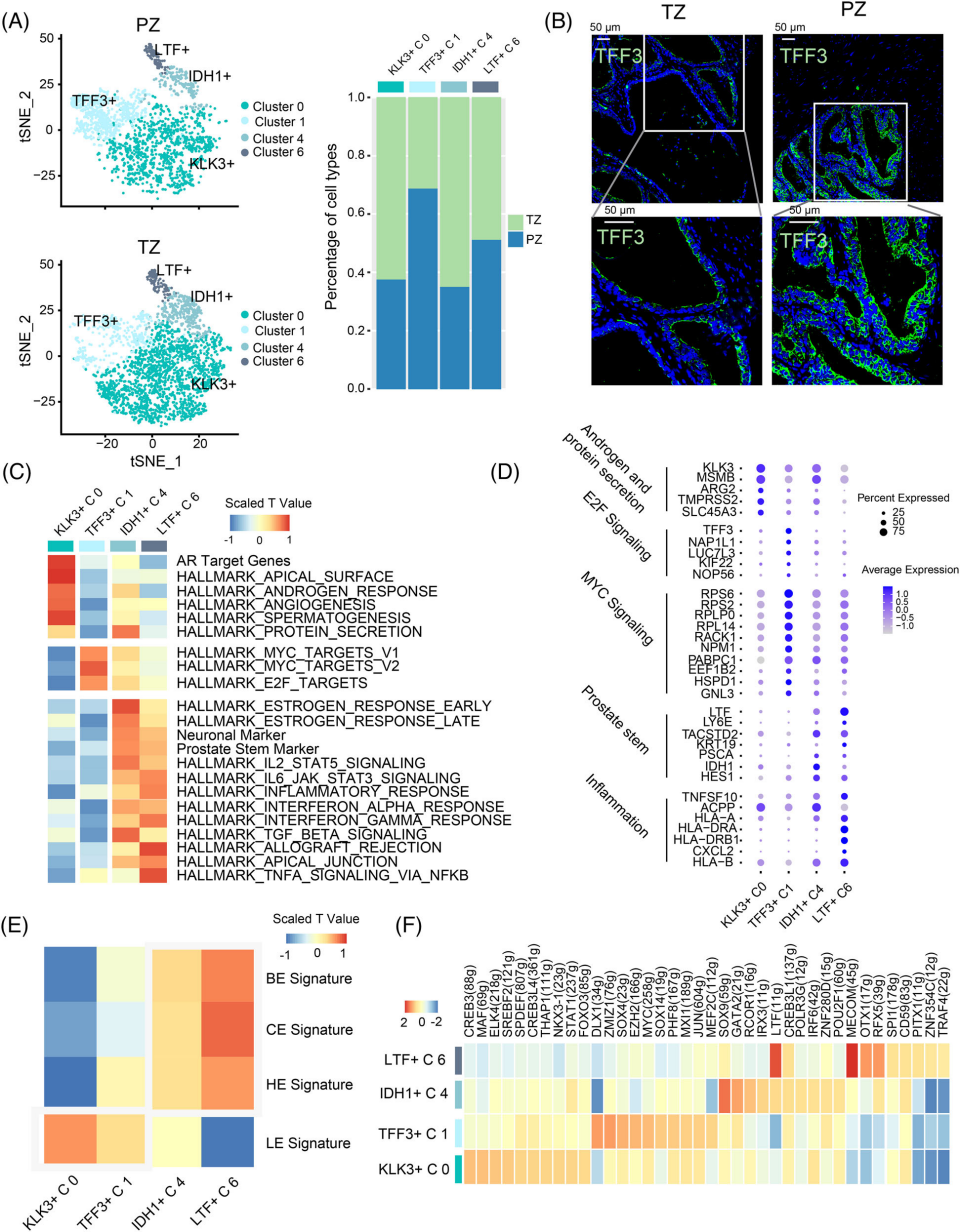
Estimated functional heterogeneity among prostate luminal cells. (A) Left: tSNE plot of distribution four luminal epithelial cells in TZ and PZ prostate tissue. Right: scRNA‐Seq data show the fraction of four luminal epithelial cells in the PZ and TZ. (B) Immunofluorescence of TFF3 in the prostate TZ and PZ (bar: 50 μm). (C) Heatmap shows differences in pathway activities scored by GSVA per cell between different luminal epithelial cells. (D) Dot plot of gene expression levels in for luminal epithelial population for selected genes. (E) Heatmap shows differences in four epithelial gene signatures scored by GSVA per cell between different luminal epithelial cells. (F) Heatmap shows the top 10 regulons in each luminal cell line. The regulon specificity score of the regulons was estimated using SCENIC

Significant heterogeneity was observed in luminal cells. Four distinct luminal sub‐clusters were observed in the aged prostates (Figures 1D–F). Spatial analysis revealed that sub‐cluster 1 luminal cells (TFF3+) were distributed in the PZ, whereas KLK3+ sub‐cluster 0 and IDH1+ sub‐cluster 4 luminal cells were enriched in the TZ (Figure 3A). Immunofluorescence confirmed that the PZ contained more TFF3+ cells than the TZ (Figure 3B). GSVA revealed that TFF3+ luminal cells had high MYC (e.g. ROCK1 and NPM1) and E2F (e.g. TFF3 and NAP1L1) pathway activity and gene expression (Figures 3C and D). KLK3+ luminal cells had high androgen and protein secretion function activity and genes (e.g. MSMB, ARG2 and TMPRSS2), which were similar to those of the traditional luminal cells (Figures 3C and D). The remaining two sub‐clusters (IDH1+ and LTF+) exhibited similar transcriptomes. Both expressed important levels of stem (e.g. IDH1, PSCA and TACSTD2) and inflammation (e.g. HLA‐A and HLA‐DRA) pathway genes (Figures 3C and D). GSVA analysis further confirms that club/hillock signatures were enriched in IDH1+ and LTF+ luminal cells (Figure 3E). These luminal cells were similar to the intermediate state between club/hillock and traditional luminal cells. The gene regulatory networks revealed that many androgen‐regulated regulons, such as SPDEF and NKX3‐1, were highly activated in KLK3+ luminal cells, whereas MYC and SOX4 regulons were activated in TFF3+ luminal cells. The club cell‐related regulon LTF was enriched in LTF+ and IDH1+ luminal cells (Figure 3F).

### 3.4 Estimated functional heterogeneity between transitional and peripheral zones

**FIGURE 4:**
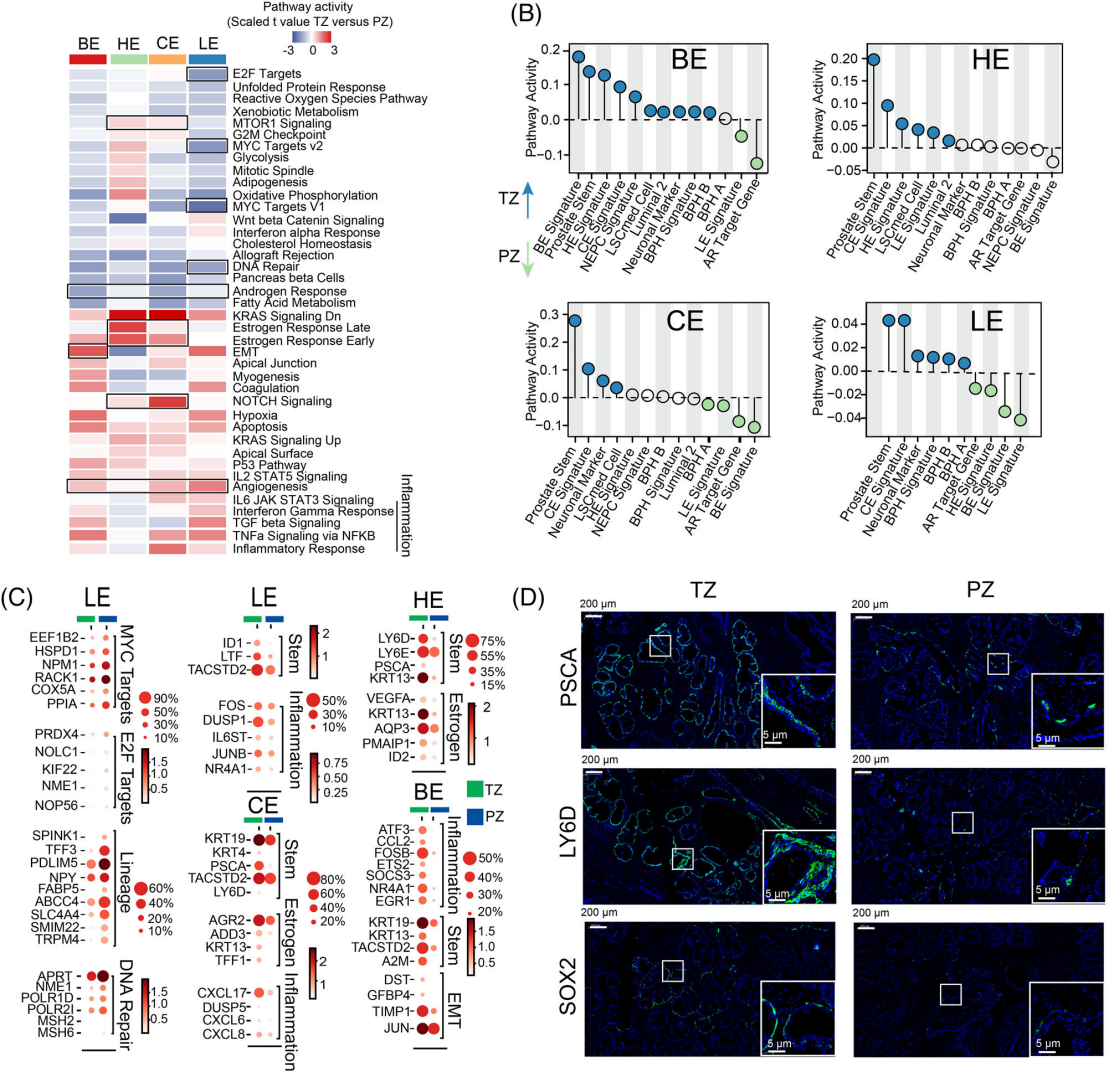
Estimated functional heterogeneity between transitional and peripheral zones. (A) Heatmap shows differences in hallmark pathway gene signatures scored by GSVA per cell between the TZ and PZ in four epithelial cells. (B) QuSAGE GSEA shows the different enrichment activities of gene sets between the PZ and TZ in four epithelial cell lines. (C) Dot plot of representative DEGs between the PZ and TZ in four epithelial cell lines. (D) Immunofluorescence of prostate sections displays expression of the prostate stem markers PSCA, LY6D and SOX2 in the TZ and PZ

We compared the functional differences between similar types of epithelial cells in the TZ and the PZ. GSVA revealed that angiogenesis‐ and inflammation‐related pathways were activated in all epithelial cells in the TZ (Figure 4A). Oestrogen, mTOR and stem/progenitor cell biology‐regulated NOTCH signalling were more activated in TZ club and hillock cells than in the PZ (Figure 4A). Both oestrogen and mTOR pathways can serve as therapeutic targets for BPH.^25^, ^29^ The E2F targets, the MYC targets and the DNA repair pathway were enriched in PZ luminal cells (Figure 4A).

Notably, compared with the PZ, the expression of stemness genes was enriched in all epithelial cells in the TZ, whereas AR and luminal‐related genes were enriched in the PZ epithelial cells (Figures 4B and C and Figure S9). The expression of some important DNA repair genes (e.g. MSH2, APRT and POLR2I), MYC and E2F pathway genes (e.g. NPM1, RACK1 and PRDX4), increased in PZ luminal cells (Figures 4A and C). Immunofluorescence assays confirmed the enrichment of stem genes (PSCA, LY6D and SOX2) in the TZ (Figure 4D).

These results suggest that the activation of stem properties is an important feature that distinguishes the TZ from the PZ and that the over‐activation of MYC, E2F and DNA repair pathways was observed in PZ luminal cells.

### 3.5 The immune microenvironment in the TZ is complex and disordered

**FIGURE 5:**
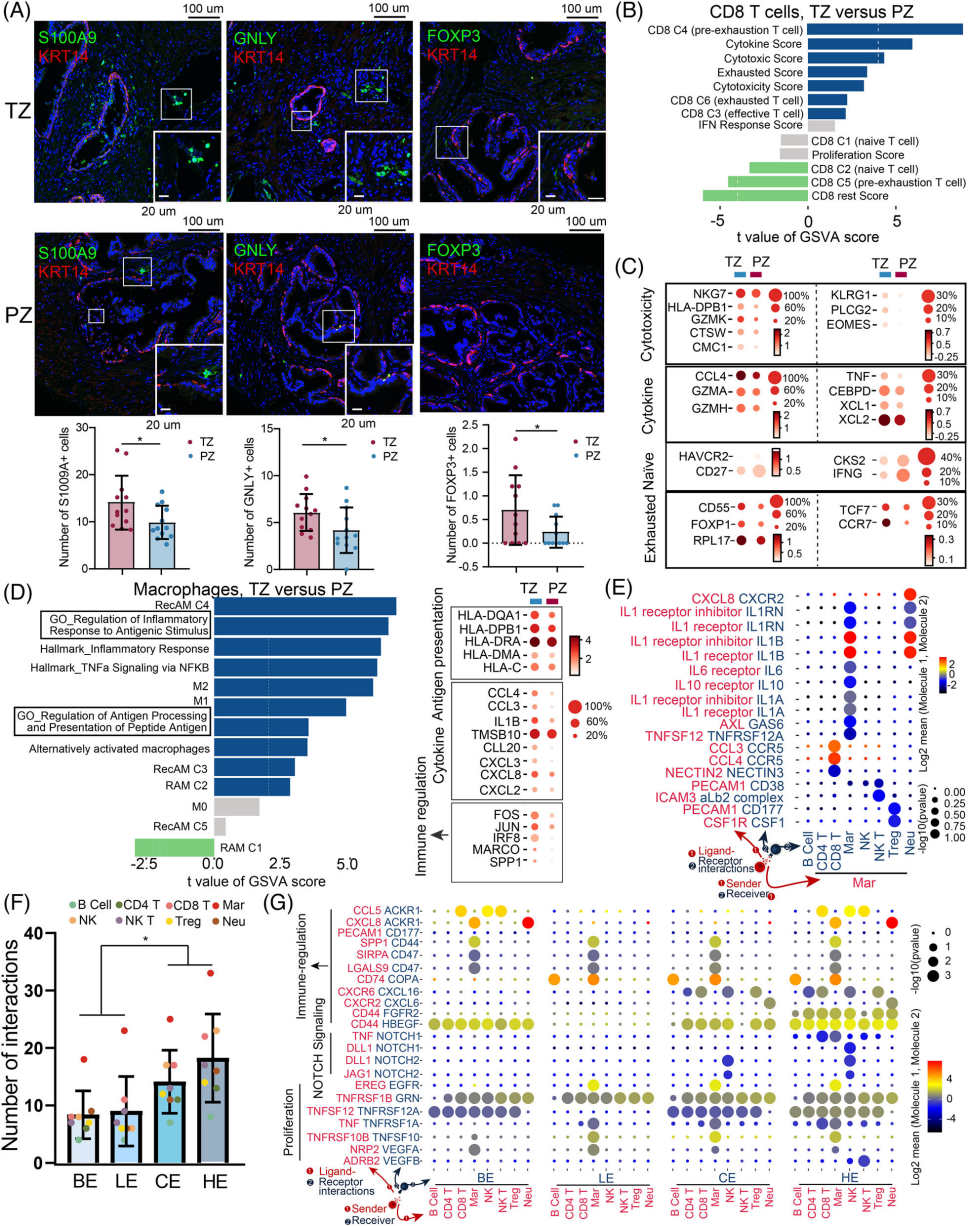
The immune microenvironment in the TZ is more complex and disordered. (A) Immunofluorescence of prostate sections displays the distribution of neutrophils (S100A9), NK cells (GNLY), Tregs (FOXP3) and basal markers (KRT14) in the prostate. Bar plot shows the number of postive cells in each TZ and PZ sections from 12 patients’ prostate gland. Error bars represent  ±  SD. *: *P* < .05. (B) Differences in pathway activities scored per cell by GSVA in CD8 T cell between the TZ and PZ; *t* values are from a linear model. (C) Dot plot of representative DEGs between the PZ and TZ in CD8 T cell. (D) Left: Differences in pathway activities scored per cell by GSVA in macrophages between the TZ and PZ; *t* values are from a linear model. Right: Dot plot of representative DEGs between the PZ and TZ in macrophages. (E) Bubble plots show unique ligand–receptor pairs between macrophages and other immune cells in the TZ. (F) Number of ligand–receptor pairs between immune cells and four epithelial cell types. The four epithelial cell types are showed in different colours. Each bar plot represents one epithelial cell types. Error bars represent  ±  SD. Coloured dots represent different immmune cell types. *: *P* < .05. (G) Bubble plots show representative ligand–receptor pairs between macrophages and other immune cells immune cells and four epithelial cell types

In the prostate immune landscape, T cells and macrophages were two major populations, whereas B cells were not commonly observed (Figure S4C). This was confirmed by immunofluorescence (Figure S10A). The difference in the proportion of immune cell types may suggest that B cell‐mediated humoral immunity is not predominant in aged prostate tissue. Both immunofluorescence and scRNA‐seq data revealed that neutrophils, NK and NK T cells (immune activation) and Treg cells (immune suppression) were enriched in the TZ (Figure 5A and Figure S10B). Although there was no difference in the distribution of T cells between the TZ and the PZ (Figure S10A), compared to those in PZ, CD8 T cells in TZ had higher cytotoxicity and exhausted scores characterised by higher expression of cytotoxicity (e.g. GZMK, NKG7, GZMA and GZMH) and exhausted (e.g. CCR7, TCF7 and CD55) genes, while naïve and rest scores and genes (e.g. CD27 and HAVCR2) were higher in PZ CD8 T cells (Figures 5B and C). Moreover, we found that previous published exhausted (CD8 C6), pre‐exhaustion (CD8 C4) and effective (CD8 C3) CD8 T cell transcriptome^30^ were more enriched in the TZ CD8 T cells (Figure 5B). In contrast, CD8 T cells in PZ were more like the naïve T cell (CD8 C1 and C2) transcriptome.^30^ Higher expression of both cytotoxicity and exhausted genes in TZ CD8 T cells suggested that TZ has a more complex and disorder immune microenvironment in which CD8 T cells are constantly being activated and then become exhausted.

Macrophage is another major immune cell clusters detected in aged prostate. Macrophages are extremely plastic and phenotypically heterogeneous immune cells, and macrophage diversity in tissues is likely not a binary division (M1 or M2 phenotype) but rather a continuum of phenotypes. In our study, macrophages can be further divided into three sub‐clusters (Figure S10C). We compared our macrophage sub‐clusters with previously published macrophage transcriptome^31^ and found that sub‐cluster 0 macrophage was similar to tissue‐resident macrophages^31^ and expressed high level of MRC1 and ITGAX (Figures S10D and E). Proliferation tissue‐resident macrophages profile^31^ and cell cycle function and genes (MKI67 and TOP2A) were enriched in sub‐cluster 2 (Figures S10D and E). For sub‐cluster 1, they were close to the pro‐inflammatory recruited macrophages profile^31^ and had high inflammation response and antigen presentation functions and genes (e.g. HLA‐B. HLA‐C, CCR7 and CD44) (Figures S10D and E). Although sub‐cluster 1 had high M1, M2, and macrophage immune‐suppressive signature enrichment (alternatively activated macrophages^32^) (Figure S10D), there is no clear M1/M2 and myeloid‐derived suppressor cells (MDSCs) separation between these clusters (Figure S10F). The expression of M1 (IL6, CD38 and TNF), M2 (MYC, IL10 and EGR2) and MDSCs (ITGAM, ARG2 and IL1B)^33^ were co‐expressed across these three macrophages clusters (Figure S10F).

Similar to the T cells, no significant difference was observed in the spatial distribution of macrophages. However, great functional heterogeneity was observed. GSVA analysis revealed that macrophages in the TZ have higher antigen presentation, TNF‐α and inflammatory pathways activities (Figure 5D). Consistently, many antigens (e.g. HLA‐DMA and HLA‐C) and cytokine (e.g. CCL3,4 and CXCL2,3,8) related genes were expressed higher in TZ macrophages (Figure 5D). More importantly, several macrophage phenotypes regulated genes including FOS/JUN which can enhance inflammatory responses of macrophages;^34^, ^35^ IRF8 favoured M1 polarisation,^34^, ^35^ and MARCO and SPP1 which have been associated with a non‐inflammatory, immune‐suppressive phenotype of macrophage activation^32^ were more abundant in the TZ (Figure 5D). Moreover, compared with those in PZ, macrophages in the TZ were more enriched in both previously described inflammation response (pro‐inflammatory recruited macrophages [RecAM C3 and C4];^31^ M1 polarisation) and suppression (alternatively activated macrophages;^32^ M2 polarisation) signatures (Figure 5D). In contrast, the macrophages in the PZ are closer to the relatively stable programming tissue‐resident macrophages (RAM C1)^31^ than those of TZ (Figure 5D). The activation of both inflammation response and suppression of macrophages shows immune disorder of TZ.

Macrophages were previously reported to cross‐present antigens to the immune cells and play a vital role in immune microenvironment regulation. Through ligand–receptor interaction analysis, we revealed a great amount of ligand–receptor interactions in both TZ and PZ (Figure S10G). The ligands secreted by macrophages could activate receptors like CD44,, SLC1A5, HAVCR2 and PTGER4 which were widely distributed on immune cells (Figure S10G). Those receptors play a key role in both immune activation (CD44^36^ and SLC1A5^37^) and suppression (PTGER4^38^ and HAVCR2^39^). There are also some unique ligand–receptor interactions that can only be observed in the TZ (Figure 5E). The macrophages can further activate and regulate CD8+ T cells through interaction with receptor CCR5 and recruit neutrophils through CXCR2. More importantly, macrophages in the TZ can be self‐regulated to pro‐ and anti‐inflammation states by IL‐1, IL‐10 (favours M1 polarisation) and IL‐6 (favours M1 polarisation) signalling (Figure 5E).

Finally, we explored the effect of immune cells on prostate epithelial cells. Previous studies have found that inflammation can promote stem features of prostate epithelial cells. Our study found that immune cells have more ligand–receptor interactions with club/hillock cells than other epithelial cell types (Figure 5F). Many proliferation‐related interactions (TNF and VEGF) were observed (Figure 5G and Figure S11). Moreover, a considerable number of immune‐regulated interactions (CXCR2/CXCL6, SIRPA/CD47 and SPP1/CD44) were observed between macrophages and epithelial cells, indicating that epithelial may play a role in immune regulation (Figure 5G and Figure S11). Notably, through secreting NOTCH agonists DLL1, JAG1 and TNF, NK cells can stimulate NOTCH signalling ligands (NOTCH2 and NOTCH4) in club/hillock cells (Figure 5G). These results indicate that NK cells may play a role in club/hillock cells’ NOTCH signalling maintenance.

### 3.6 Fibroblasts enriched in the TZ might support epithelial proliferation and immune cell recruitment

**FIGURE 6:**
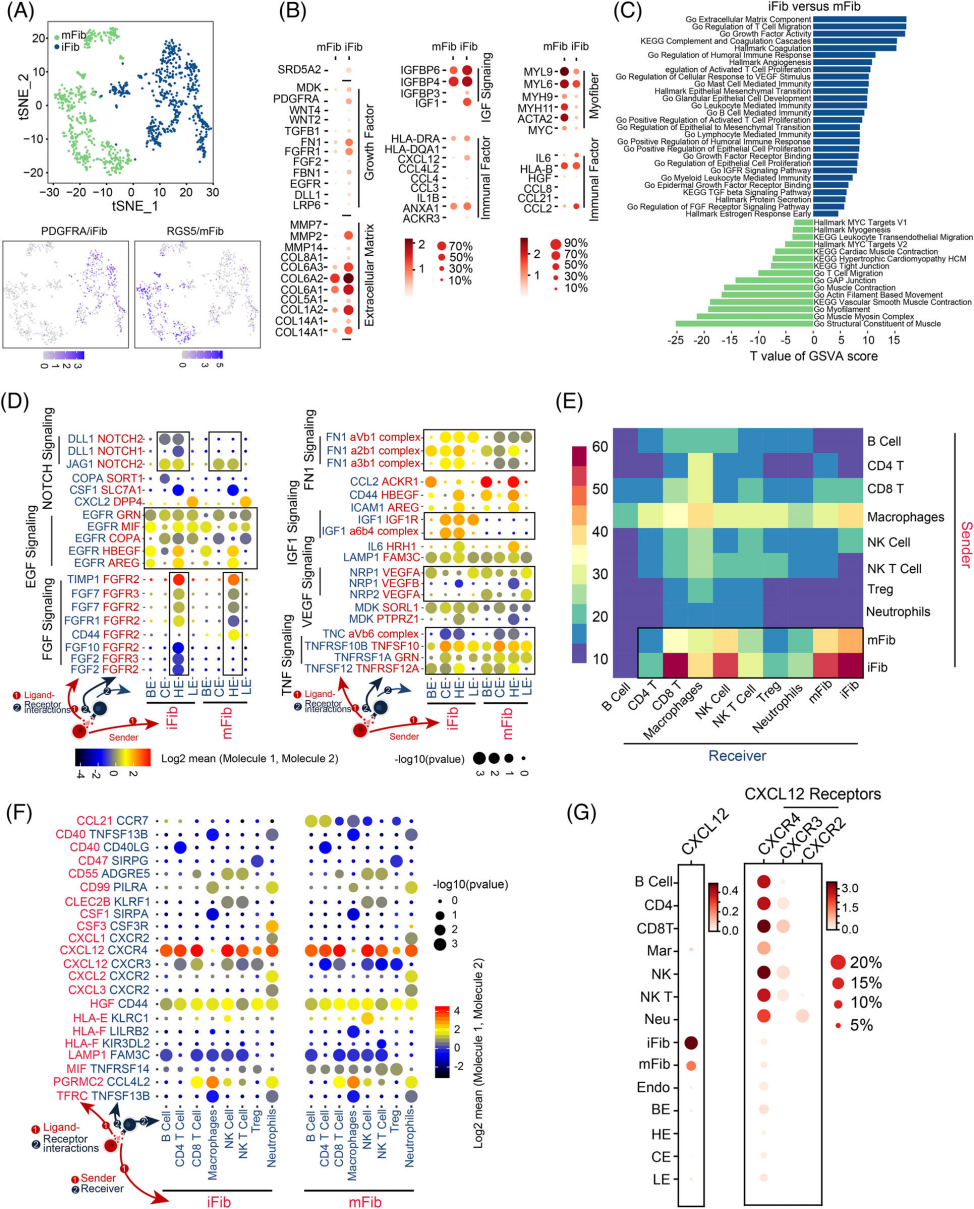
Fibroblasts enriched in the TZ might support epithelial proliferation and immune cell recruitment. Upper: t‐SNE plot of fibroblasts. Fibroblasts were divided into two clusters (indicated by colours). The bar plot shows the fraction of two types of fibroblasts in the TZ and PZ. Down: Expression of iFib (PDGFRA) and mFib (RGS5) marker genes in fibroblasts. (B) Dot plot of represented DEGs between iFib and mFib. (C) Differences in hallmark pathway gene signature activities scored per cell by GSVA between iFib and mFib. Shown are *t* values from a linear model. (D) Bubble plots show ligand–receptor pairs between iFib and mFib in epithelial cells. (E) Heatmap showing the number of potential ligand–receptor pairs between cell groups predicted by CellphoneDB 2. (F) Bubble plots show ligand–receptor pairs of immune cytokines between iFib and mFib and immune cells. (G) Dot plot shows expression of CXCL12 and its receptors (CXCR4, CXCR3 and CXCR2) across different cell types

We found two types of fibroblasts, iFib (PDGFRA+) and mFib (RGS5+), in prostate tissues (Figure 6A). Consistent with a previous study,^32^ we found that iFib had enriched expression of IL6, and chemokines such as CCL3, CCL2 and CXCL12 and mFib expressed elevated levels of α‐SMA (ACTA2). Enrichment and differentially expressed genes analyses revealed that ECM, growth factor secretion and IGF‐1 pathways and genes were significantly enriched in iFib. Muscle fibre‐related pathways and genes were activated by mFib. Both fibroblast types secreted various immune‐related factors (Figures 6B and C). Immunofluorescence assays and scRNA‐Seq data revealed that iFib, mFib and SRD5A2 expression was enriched in the TZ (Figure S12A). Compared with the PZ, both fibroblasts were significantly activated in almost all biological functions in the TZ (Figure S12B), particularly pathways and genes related to immunity (CXCL12, IL6 and SOCS3) and proliferation (NRP1,2 and FGF10) (Figures S12B and C). In particular, the expression of NOTCH signalling ligands DLL1 and JAG1 was also significantly high in TZ fibroblasts (Figure S12C).

Through ligand–receptor interaction analysis, we observed a large amount of outgoing and incoming activity in the fibroblasts (Figure S12D). Fibroblasts in the TZ had more types of ligand–receptor interactions with other cells than those in the PZ (Figures S12D, E). In fibroblast–epithelial interactions, we found common proliferation pathways such as TNF, EGF and FGF (Figure 6D). Fibroblasts express the secreted NOTCH agonists DLL1 and JAG1, which can stimulate NOTCH signalling ligands (NOTCH1 and NOTCH2) in club/hillock cells by creating a localised signalling niche around these cells in the TZ (Figure 6D).

Fibroblasts showed the strongest interaction with immune cells (Figure 6E). They expressed elevated levels of CXCL, CCL, HLA and CSF family cytokines, particularly CXCL12, the receptors of which are extensively expressed on immune cells (Figures 6F and G).

## 4 DISCUSSION

The prostate is a heterogeneous gland, and the cellular and functional heterogeneity between the TZ and PZ tissues may be one of the reasons for the spatial distribution of prostate diseases. Using single‐cell sequencing technology, Strand lab determined the cellular composition and distribution of normal prostate from young donors, revealing two new types of epithelial cells, namely ‘club’ and ’hillock’. ^15^ Further studies clarified the stem‐ and castration‐insensitive properties of these two types of cells.^28^ Moreover, two independent studies observed luminal to club cell transition in human prostate tissues during 5‐alpha‐reductase inhibitor^40^ and androgen deprivation therapy,^24^ respectively. Our study further confirmed androgen‐independent and neural stem features of club/hillock cells. The scRNA‐seq and immunofluorescence results indicate that club/hillock cells have low androgen pathway activity and loss of androgen receptor expression but express significantly high stem and neuroendocrine genes. McNeal proposes BPH as ‘embryonic reawakening’ and speculates this ‘embryonic reawakening’ may drive epithelial and stoma hyperplasia leading to BPH.^41^ Many studies have recently found that mesenchymal and epithelial stem cells play an essential role in the development, progression and therapeutic resistance of BPH.^42^, ^43^ Therefore, we speculate that stem‐featured and androgen‐independent club/hillock cells may survive under androgen‐deprived conditions and provide stem‐like niches to regenerate other epithelial cells or PCa cells. The presence of club/hillock cells may be one of the reasons for the failure of anti‐androgen therapy for BPH and PCa.

In addition, elevated NOTCH pathway receptor (NOTCH1 and NOTCH2) expression and NOTCH signalling activities were observed in club/hillock cells. Moreover, club/hillock cells were primarily found in the TZ than in PZ, and those cells in the TZ had stronger NOTCH pathway activity. NOTCH signalling is the driving force in regulating stem/progenitor cell biology in various tissues,^44^, ^45^ leading us to determine the source of the ligands that activate NOTCH receptors in club/hillock cells. Ligand–receptor interaction analysis revealed significant NOTCH ligand–receptor interactions (DLL1/NOTCH1, DLL1/NOTCH2 and JAG1/NOTCH2) from fibroblasts and NK cells to club/hillock cells. Notably, compared with that in PZ, the expression of NOTCH signalling ligands DLL1 and JAG1 and distribution of fibroblasts and NK cells were significantly higher in TZ, thus creating a localised NOTCH signalling niche around the TZ club/hillock cells. This may be important for maintaining the stemness of the club/hillock cells. These insights suggest that novel prostate disease therapies targeting club/hillock cells (e.g. targeting NOTCH) may be useful in combination with androgen blockers.

We observed greater heterogeneity in the aged luminal population than Henry et al.’s data from young donors.^15^ In addition to the traditional KLK3+ luminal cells, we annotated the TFF3+ luminal subtypes in aged prostates. This luminal subtype featured higher MYC and E2F pathway activities and was more enriched in the PZ than in TZ. Moreover, by comparing the differences in the functional enrichment of luminal cells between the TZ and PZ, we found that luminal cells in the PZ expressed higher levels of MYC and E2F target genes, androgen/lineage and DNA repair genes. Excessive activation of the MYC and E2F pathways and their regulated genes have been considered to be an important event in the malignant degeneration of prostate luminal cells,^46^, ^47^ which may be one of the reasons why luminal cells in the PZ are more susceptible to malignancy.

The other two types of LTF+ and IDH1+ luminal subtypes exhibit similar transcriptomes. These subtypes express both luminal and club/hillock‐cell markers. They were more similar to the intermediate cell types between club/hillock and traditional luminal cells. Song et al.^48^ also found that the LTF+ luminal subtype was more prevalent in PCa tissues than in normal tissue. Therefore, they called LTF+ luminal cells cancer‐associated club cells. As luminal to club cell translation has been observed in recent studies,^24^, ^40^ we believe that LTF+ luminal cells are unstable intermediate cells and that under the stimulation of tumour cells, more of these intermediate cells may be induced. This may result in higher levels of LTF+ luminal cells in tumour tissues than in normal tissues. The role of LTF+ luminal cells in PCa and prostate disease development and progression warrants further investigation.

Fibroblasts play a significant role in the organisation and maintenance of the tissue environment. Both Joseph et al.^28^ and our study observed proximal‐distal fibroblast density differences in human prostates in prostate tissues. Joseph et al. observed that peri‐epithelial fibroblasts express secreted WNT inhibitors, namely SFRPs and DKK1, which could serve as a buffer against stromal WNT ligands by creating a localised signalling niche around individual prostate glands. In our study, we found that proximal fibroblasts not only play a significant role in regulating epithelial cell function, but fibroblasts in the TZ also exhibit stronger immunity and proliferation functions and have more ligand–receptor communication with epithelial and immune cells, compared with those in PZ. This may amplify the critical role of fibroblasts in the recruitment and proliferation of immune and epithelial cells in the TZ. The higher density and stronger functions of fibroblasts in the TZ, compared to those in PZ, could help to create a stem niche and a more complex immune microenvironment associated with the TZ.

**FIGURE 7:**
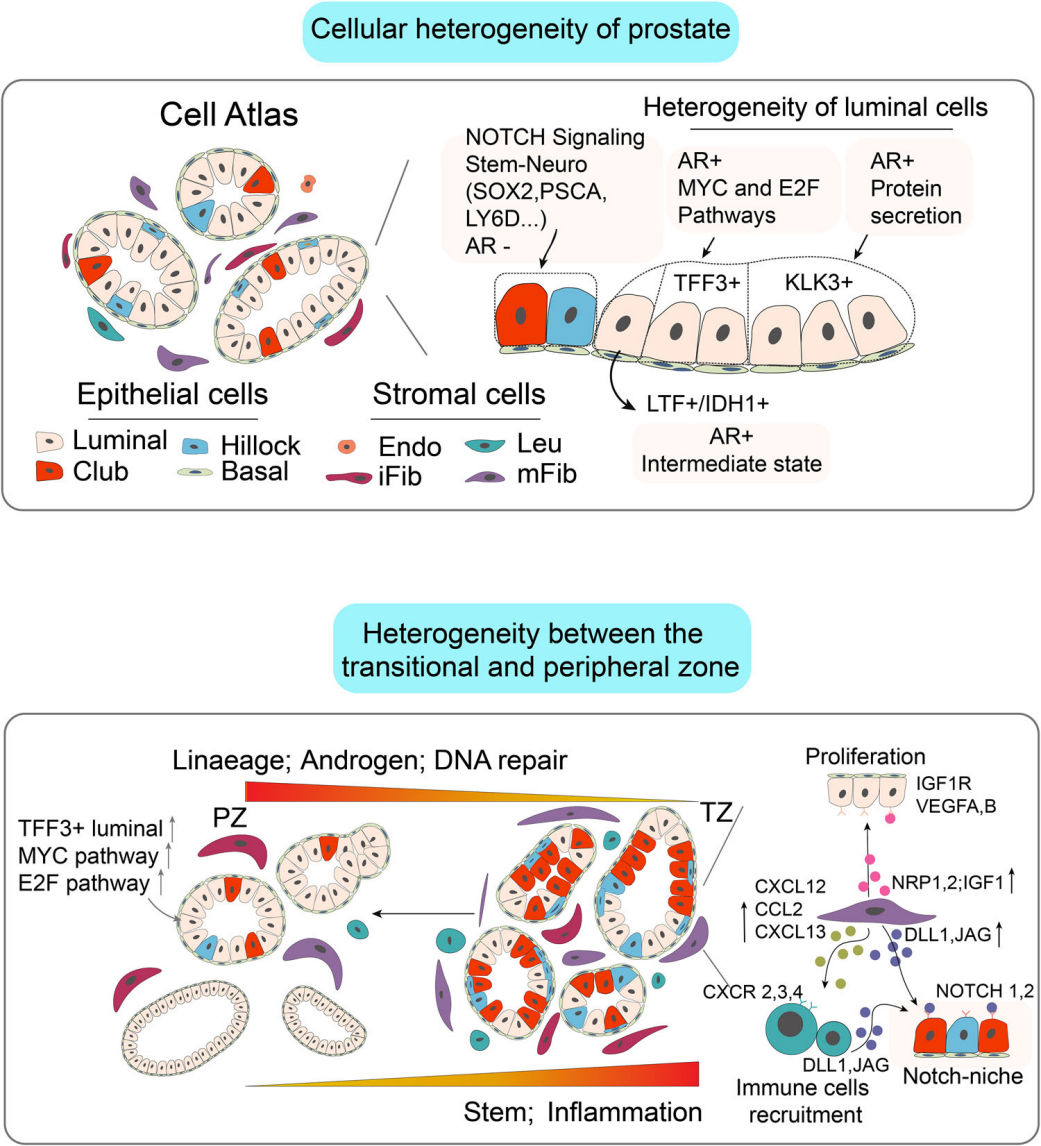
Summary of the main findings of our study. Upper: Shows the cellular heterogeneity of prostate and major cell types in the prostate. In epithelial cells: club/hillock cells were featured by high NOTCH signaling activity, Stem‐Neuro properties but low androgen activity; Great heterogeneity was observed in the luminal cells. KLK3+ luminal cells were traditional functional luminal cells with high androgen activity; TFF3+ luminal cells featured by high MYC and E2F pathways activities; LTF+/IDH1+ were intermediate cells between luminal and club/hillock cells. Down: Heterogeneity between the TZ and PZ. Club/hillock cells were more distributed in the TZ, whereas TFF3+ luminal cells were more distributed in the PZ. The expression of stem and inflammatory pathways and genes decreased gradually but that androgen, lineage and DNA repair pathways and genes increased from the TZ to the PZ. In the immune landscape, we found that the immune microenvironment in the TZ is more complex and disordered with more infiltration of NK and Treg cells. TZ has higher fibroblast density and fibroblast in TZ has stronger functions. The TZ fibroblasts provide NOTCH‐niche around the club/hillock cells and through ligand/receptor communication, they also play an important role in epithelial cells’ proliferation and immune cell recruitment

Chronic inflammation has long been implicated in BPH development and progression.^49^, ^50^, ^51^ Mucosal immune disorders are frequently observed in many BPH patients, and patients with higher baseline inflammation display greater prostate volume, higher risk of urinary retention and higher risk of symptomatic progression.^49^, ^50^, ^51^, ^52^ Moreover, inflammation status is positively correlated with loss of luminal AR expression^53^ and anti‐androgen therapy resistance. In addition, inflammation could potentially activate the stem feature of the prostate microenvironment by inducing luminal to club adaption.^40^ In our study, great function heterogeneity and immune activation and suppression disorder were observed in the TZ immune microenvironment. There is considerable ligand/receptor interaction between immune cells and club/hillock cells. Notably, NK cells induced the club/hillock club/hillock cells’ NOTCH signalling activation may be one of the reasons for inflammation induced stem feature transformation in the TZ. The activation of stem properties, inflammatory infiltration and loss of androgen activity are key features that distinguish the TZ from the PZ, which may contribute to TZ hyperplasia. Figure 7 summarises the major findings of our study.

The major limitation of our study was the sample and tissue selection, although we ensured that our selected samples were not influenced by the tumour, prostate intraepithelial neoplasia, chronic and acute inflammatory lesions or BPH nodules with glandular tissue. It is still difficult to obtain completely normal samples from aged prostates. This is also one of the dilemmas of current research on aged prostate tissues. It would be more conclusive if future studies could perform the analysis using autopsy prostate tissues from both young and aged men. We hope that the present findings obtained through big data analysis can provide novel insights for future functional research that may establish a foundation for new drug and target discovery.

## 5 CONCLUSIONS

In summary, our study successfully clarifies the heterogeneity between the TZ and PZ of aged prostate and helps elucidate the possible mechanisms underlying the spatial distribution of prostate diseases.

## CONFLICT OF INTEREST

The authors declare no potential conflicts of interest.

## Supporting information

Supporting Information

Supporting Information

Supporting Information

Supporting Information

Supporting Information

Supporting Information

Supporting Information

Supporting Information

Supporting Information

Supporting Information

Supporting Information

Supporting Information

Supporting Information

Supporting Information
