# Supplementary material for: Single‐cell RNA‐sequencing technology demonstrates the heterogeneity between aged prostate peripheral and transitional zone
Source: Clin Transl Med. 2022 Oct 17;12(10):e1084. doi: 10.1002/ctm2.1084 (PMC9574492; doi:10.1002/ctm2.1084)
Supplement: Supplementary file 1 — Supporting Information [file CTM2-12-e1084-s007.docx]

**Supplementary Materials and Methods**

Evaluation of immunostaining

The slides were evaluated by observers who were blinded to the origin of the samples using an inverted Olympus microscope with the assistance of image processing software: Image Pro Plus 6.0 software (Media Cybernetics Inc). The S100A9 and GNLY, FOXP3, MS4A1, CD3D and CD68 positive individual infiltrating cells were quantified as the numbers of positive cells per field (200X). When scoring Ki67 and PCNA positive cells we calculate the percentage of positive cells out of all cells in each field (200X). H-score was employed to measure the RGS5 and PDGFRA staning and calculated using the following formula: (percentage of areas of weak intensity × 1) + (percentage of areas of moderate intensity × 2) + (percentage of areas of strong intensity × 3). As a consequence, H-score provided a semi-continuous score between 0 and 300 for each core. For all samples we randomly count 5 field of views for each sample and calculate the mean value.

Single-cell sequencing and data analysis

*Single cell sequencing Mapping*

For 10× Genomics platform, the raw reads were mapped to the human reference genome (build GRCh38 with ensemble version 92 gene annotation) with CellRanger V 3.1.0 (10× Genomics).

For Singleron platform, the raw reads were processed to generate gene expression profiles using an internal pipeline. In brief, after filtering one read without polyT tails, the cell barcode and UMI were extracted. Adapters and polyA tails were trimmed (fastp V1) before aligning to GRCh38 with ensemble version 92 gene annotation (fastp 2.5.3a and featureCounts 1.6.2)^1^. Reads with the same cell barcode, UMI and gene were grouped to calculate the number of UMIs per gene per cell. The UMI count tables of each cellular barcode were used for further analysis.

*Computational identification of doublets*

We identified doublets using the R package DoubletFinder (v2.0.3)^2^. The ‘paramSweep_v3’ and ‘summarizeSweep’ functions were used with default settings to determine the ‘pK’ value, which defines the principal component neighborhood size of real artificial data. The proportion of artificial doublets (the ‘pN’ value) was set to 0.25. The proportion of homotypic doublets was identified using the ‘modelHomotypic’ function with gene-based lineage annotations for each cluster (from an overclustered resolution of 0.2). Singlet versus doublet classifications were obtained using the ‘doubletFinder_v3’ function with two settings. First, ‘nExP’ was set to the expected doublet rate according to the Chromium Single Cell 3’ v2 reagent kit user guide (10x Genomics). Second, this rate was adjusted to the proportion of homotypic doublets. Cells classified as a doublet with either setting were assigned a doublet designation.

*Stress removal*

Cells displaying high-stress signatures and high percentages of mitochondrial content (% mito > 25%) were removed. To identify cells with a high stress signature, principal component analysis was used as described previously^3^. In brief, principal component analysis was performed on the expression level of a list of prostate-specific stress response genes (M10970)^4^. The cells’ projection to the first principal component was used as a “stress score”. Highly stressed cells were chosen as the 5% of cells that had the top ‘stress score’. Clusters (from an overclustered resolution of 1) were removed if at least 50% of the cells were identified as highly stressed. The remaining cells were subsequently reclustered, and new t-SNE plots were generated.

*Integrated and Clustering analyses*

We used Seurat (v 3.5.1)^5^ to create data objects from the matrix outputs. Low-quality cells were filtered out by consecutively filtering each sample individually based on UMI counts and the number of genes. Genes expressed in five cells or less were filtered out along with cells expressing fewer than 200 unique genes. Low-quality cells and multiplets were excluded by removing cells with fewer than 200 unique genes and greater than 2500 unique genes. Data were normalized to log scale using the ‘NormalizeData’ function with a default scale parameter of 10000. The top 2000 highly variable genes were identified using the ‘FindVariableFeatures’ function with the “vst” method with default parameters. Mitochondrial and ribosomal genes were removed if they were highly variable. Cells from the six samples were then integrated using canonical correlation analysis (CCA) with highly variable genes. The intersection of these genes between the six samples was used to calculate 50 CCAs, and the first 30 were aligned. These 30 aligned CCAs were used for t-SNE visualization and clustering.

The effects of variation in sequencing depth were regressed out by including ‘nUMI’ as a parameter in the ‘ScaleData’ function. Scaled highly variable genes were used as input for PCA using the ‘RunPCA’ function. The first 15 principal components (PCs) and a resolution of 0.2 were used for clustering using ‘FindClusters’. T-SNE was applied for two-dimensional representation of the first 15 PCs with ‘RunTSNE’.

To verify the reliability of CCA integration, we also used Harmony^6^ to integrate the data, and compared the cell clustering results with CCA.

*CNV analysis*

After quality control was performed and stress and doublet cells were removed we integrated our data with those of Henry et al.^7^ and Ma X et al.^8^, we used their data as a benign (Henry et al) and malignant (Ma X et al) reference respectively. Initial CNVs were estimated by the expression levels of genes within each chromosome region using inferCNV R package^9^. The CNVs of cells were calculated by expression level from scRNA-seq data for each cell with a cutoff of 0.1. The relative expression values of analyzed genes were limited to [−1,1]. We considered cells apart from Henry et al^7^ (sample D27PY) to be non-malignant cells and used their average estimated CNV as background. The CNV score of each cell was calculated as quadratic means of (CNV region -1).

*Pathway or gene set enrichment analysis*

An expression matrix derived from the data slot of the Seurat object was used for enrichment analysis. Quantitative set analysis for gene expression (QuSAGE) gene set enrichment analysis (GSEA)^10^ and gene set variation analysis (GSVA)^11^ were utilized to perform gene set enrichment-type analysis comparing with publicly available prostate gene sets. H hallmark gene sets and C2 CP:KEGG gene sets were downloaded from MSigDB v 6.2 at <http://software.broadinstitute.org/gsea/msigdb/genesets.jsp>. Reactome pathway gene sets were downloaded from the reactome database at <https://reactome.org/dev/graph-database>. Prostate-specific gene signatures and literature sources are summarized in Supplementary Table 3 and only the gene upregulated in a given subtype of cells were used for the enrichment analysis. Due to same genes in gene sets used in QuSAGE GSEA analysis are expressed by a very low number of cells. This will bias the result of QuSAGE GSEA analysis. To further validation the QuSAGE GSEA analysis results we perform the pseudo-bulk (Analyze specific cell type in a sample as a whole by summing all cell counts of this cell type) GSEA analysis. For GSVA analysis, significantly different enrichment scores across groups were determined by the FDR-corrected p value < 0.05 using the R package “limma”^12^.

*Epithelial cell type identification*

The tool SingleR^13^ (version 1.0.0) was employed to identify epithelial cell types. Our epithelial cells were cluster into 8 clusters. We used the epithelial cell type expression matrix from Henry, G.H. et al.’s data as a reference to identify epithelial cell types.

*DEG calculation*

Differentially expressed genes (DEGs) were calculated by using the “FindMarkers” function (“wilcox” method) implemented in Seurat. Differentially expressed genes were identified as follows: 1) genes present in at least 25% of the cell cluster; 2) absolute value of Log2-fold change greater than 0.25; 3) Bonferroni-corrected p value < 0.05.

*Interaction between cell types*

We extracted the expression count matrix of each cell lineage from the Seurat object together with cell type metadata. These data were used as input for cellphonedb (release 0.0.6) ^14^ with the ‘statistical analysis method’ in Python (v2.7.13). This analysis was employed to identify significant interactions (p < 0.05) between receptors and ligands against a null distribution using a curated receptor-ligand database.

*Regulome analysis*

To understand gene regulatory networks in each cell, we used SCENIC (v1.1.2-2)^15^ together with dependencies AUCell (v1.8.0), Rcistarget (v1.6.0) and Genie3

(v1.8.0). An expression matrix from the data slot of the Seurat object of interest was used as input for the ‘initializeScenic’ function together with cistarget databases ‘hg38_refseqr80_500 bp_up_and_100 bp_down_tss.mc9nr.feather’, “hg38_regseq-r80_10 kb_up_and_down_tss.mc9nr.feather’ downloaded from <https://resources.aertslab.org/cistarget/>. The expression matrix was filtered for genes expressed with greater than 3 UMIs in 1% of cells and genes detected in at least 1% of cells. Positive and negative associations were identified by determining Spearman correlation on the expression matrix. Regulators were identified using the ‘runGenie3’ function with default parameters. Gene regulatory networks were scored using default parameters in ‘runSCENIC_1_coexNetwork2 modules’, ‘runSCENIC_2_createRegulons’ and ‘runSCENIC_3_scoreCells’. Regulon activity was evaluated for each cluster by averaging the AUC values per cell. To quantify the cell-type specificity of a regulon, we calculated the regulon specificity score (RSS) in each cluster, as described in a previous study^16^. When finding significantly different regulons across groups, we employed the R package “limma”^12^ to calculate the fold change of AUC values of each regulon across groups, and FDR-corrected p values < 0.05 were considered to be significant.

*Trajectory analysis*

Single-cell gene expression trajectories were constructed using Moncole (v2.14.0)^17^ by importing Seurat data using the ‘importCDS’ function. Size factors and dispersion values were calculated using ‘estimateSizeFactors’ and ‘estimateDispersions’ functions, respectively. Highly variable genes were detected by using the “dispersionTable” function and filtered by using a threshold of 0.1 mean expression. These genes were utilized as input for the ‘setOrderingfilter’ function; dimensionality reduction was conducted using the ‘reduceDimension’ function with the DDRTree method, and cells were placed onto a pseudotime trajectory using ‘orderCells’.

*RNA Velocity*

We used the velocyto.py^18^ annotator for each mapped BAM file with the default parameters for 10x Genomics technology and the gencode hg38_rmsk gtf file for annotation. The resulting loom object for each sample was loaded and processed in R using the Velocyto. R (v. 0.17) package. We used the embedding from Monocle DDRTree representation for cell-cell distance calculation and final velocity plots. The estimation of RNA velocity was performed with the grouping of 10 cells; other parameters were set to the default.

References

1. Liao Y, Smyth GK, Shi W. featureCounts: an efficient general purpose program for assigning sequence reads to genomic features. Bioinformatics. 2014; 30(7): 923-30.
2. McGinnis CS, Murrow LM, Gartner ZJ. DoubletFinder: Doublet Detection in Single-Cell RNA Sequencing Data Using Artificial Nearest Neighbors. Cell Syst. 2019; 8(4): 329-337.
3. Henry GH, Malewska A, Joseph DB, Malladi VS,. A Cellular Anatomy of the Normal Adult Human Prostate and Prostatic Urethra. Cell Rep. 2018; 25(12): 3530-3542.
4. Liberzon A, Subramanian A, Pinchback R, Thorvaldsdo´ttir H, Tamayo P, Mesirov JP. Molecular signatures database (MSigDB) 3.0. Bioinformatics. 2011. 27(12): 1739-1740.
5. Butler A, Hoffman P, Smibert P, Papalexi E, Satija R. Integrating single-cell transcriptomic data across different conditions, technologies, and species. Nat Biotechnol. 2018; 36(5): 411-420.
6. Korsunsky I, Millard N, Fan J,et al. Fast, sensitive and accurate integration of single-cell data with Harmony. Nat Methods. 2019; 16: 1289-1296.
7. Henry GH, Malewska A, Joseph DB, et al. A Cellular Anatomy of the Normal Adult Human Prostate and Prostatic Urethra. Cell Rep. 2018; 25(12): 3530-3542.doi: 10.1016/j.celrep.2018.11.086.
8. Ma X, Guo J, Liu K, et al. Identification of a distinct luminal subgroup diagnosing and stratifying early stage prostate cancer by tissue-based single-cell RNA sequencing. Mol Cancer. 2020; 19(1): 147.doi: 10.1186/s12943-020-01264-9.
9. Patel AP, Tirosh I, Trombetta JJ, et al. Single cell RNA-seq highlights intratumoral heterogeneity in primary glioblastoma. Science. 2014; 344(6190): 1396 -1401.
10. Yaari G, Bolen CR, Thakar J, Kleinstein SH. Quantitative set analysis for gene expression: a method to quantify gene set differential expression including gene-gene correlations. Nucleic Acids Res. 2013; 41(18): e170.
11. Hänzelmann S, Castelo R, Guinney J. GSVA: gene set variation analysis for microarray and RNA-seq data. BMC Bioinformatics. 2013; 14: 7.
12. Ritchie ME, Phipson B, Wu D, et al. “limma powers differential expression analyses for RNA-sequencing and microarray studies” . Nucleic Acids Res. 2015; 43(7): e47.
13. Aran D, Looney AP, Liu L, et al. “Reference-based analysis of lung single-cell sequencing reveals a transitional profibrotic macrophage”. Nat Immunol. 2019; 20(2): 163-172.
14. Efremova M, Vento-Tormo M, Teichmann SA, Vento-Tormo R. CellPhoneDB: inferring cell–cell communication from combined expression of multi-subunit ligand–receptor complexes. Nat Protoc. 2020; 15(4): 1484-1506.
15. Aibar S, González-Blas CB, Moerman T, et al. SCENIC: single-cell regulatory network inference and clustering. Nat Methods. 2017; 14(11): 1083-1086.
16. Suo S, Zhu Q, Saadatpour A, Fei L, Guo G, Yuan GC. Revealing the Critical Regulators of Cell Identity in the Mouse Cell Atlas. Cell Rep. 2018; 25(6): 1436-1445.
17. Qiu X, Mao Q, Tang Y, et al. Reversed graph embedding resolves complex single-cell trajectories. Nat Methods. 2017; 14(10): 979-982.
18. La Manno G, Soldatov R, Zeisel A, et al. RNA velocity of single cells. Nature. 2018; 560(7719): 494-498.
