# Supplementary figures and images for: Single‐cell RNA‐sequencing technology demonstrates the heterogeneity between aged prostate peripheral and transitional zone
Source: Clin Transl Med. 2022 Oct 17;12(10):e1084. doi: 10.1002/ctm2.1084 (PMC9574492; doi:10.1002/ctm2.1084)

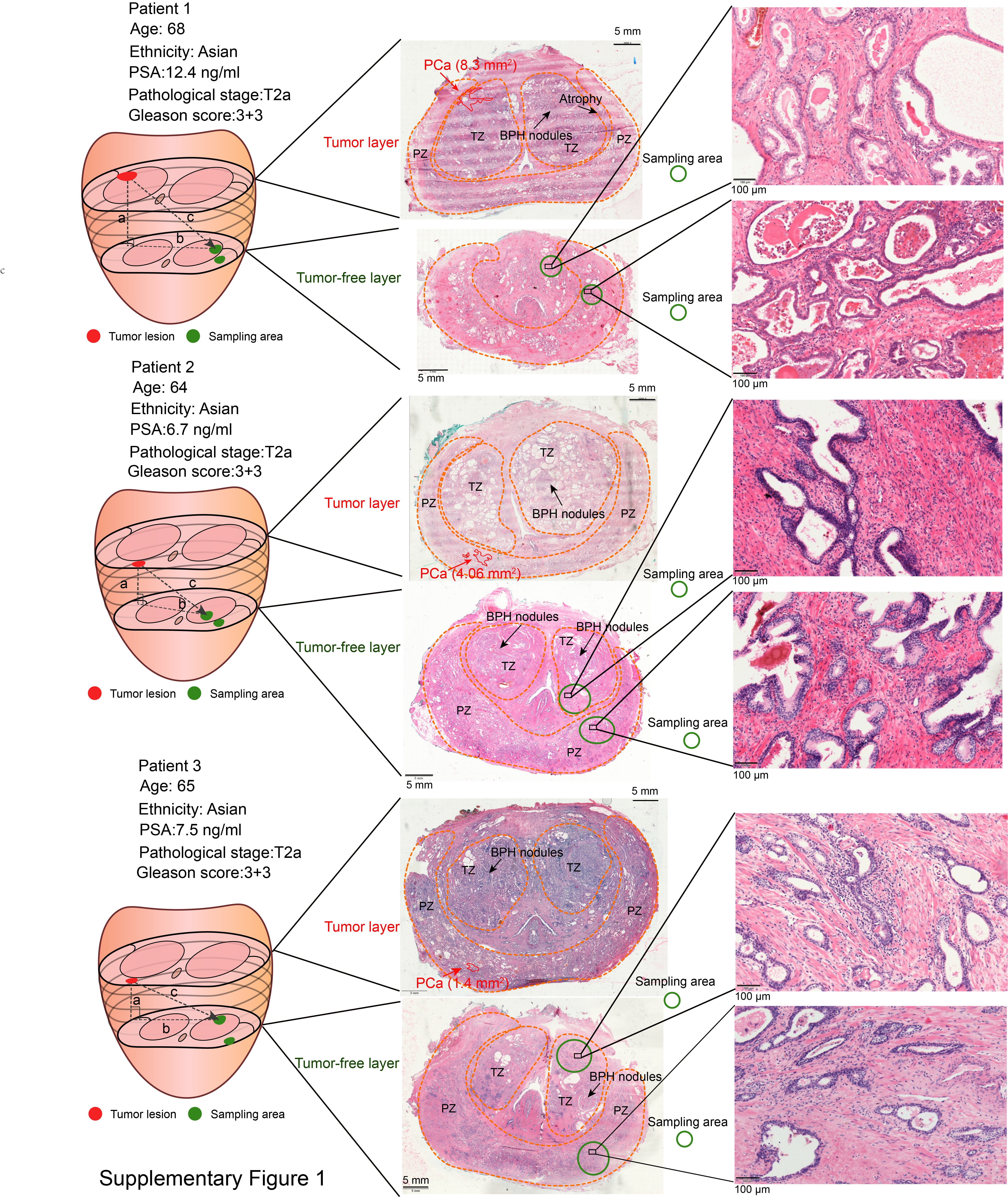

Supplement: Supplementary file 2 — Supporting Information [file CTM2-12-e1084-s009.png]

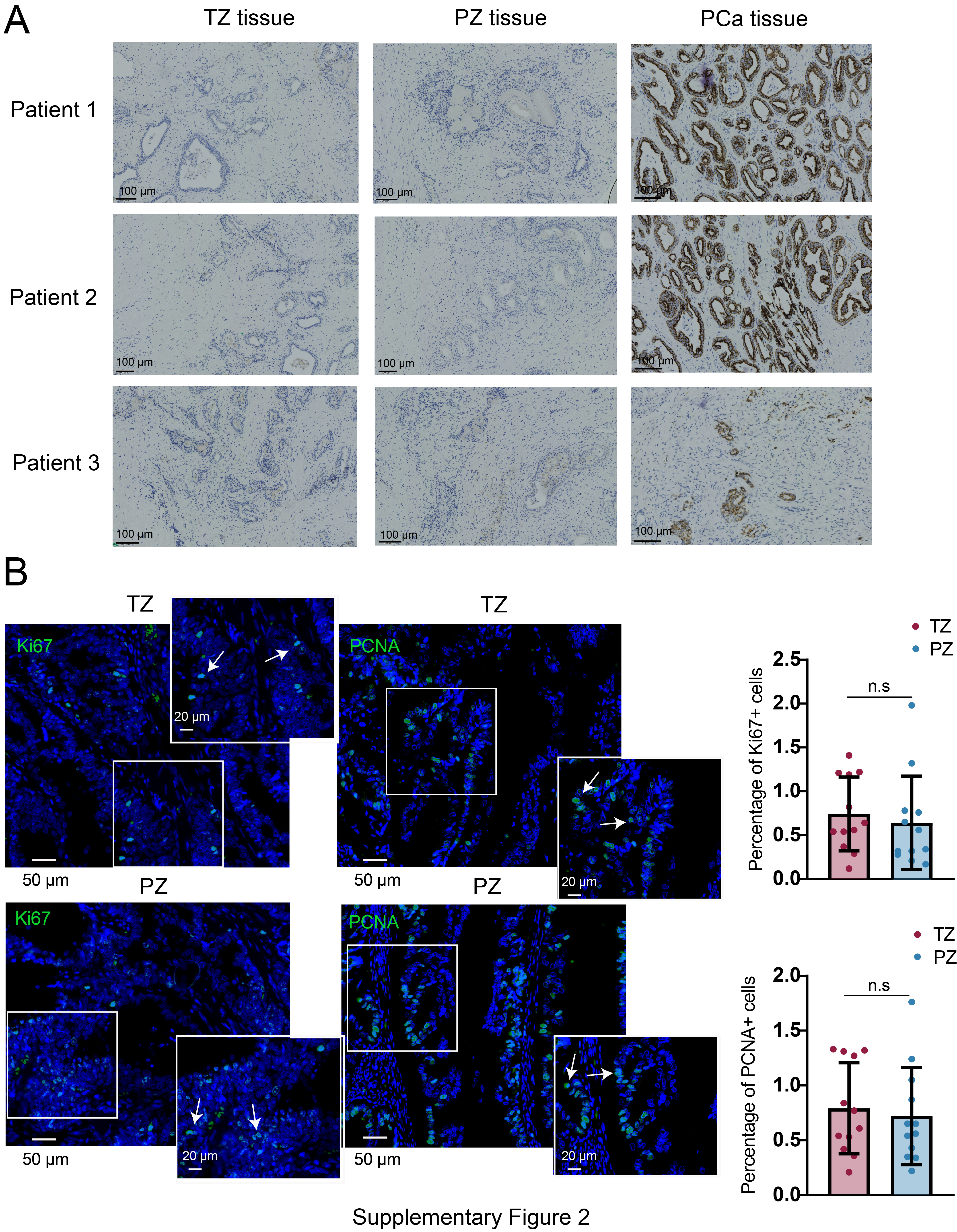

Supplement: Supplementary file 3 — Supporting Information [file CTM2-12-e1084-s013.png]

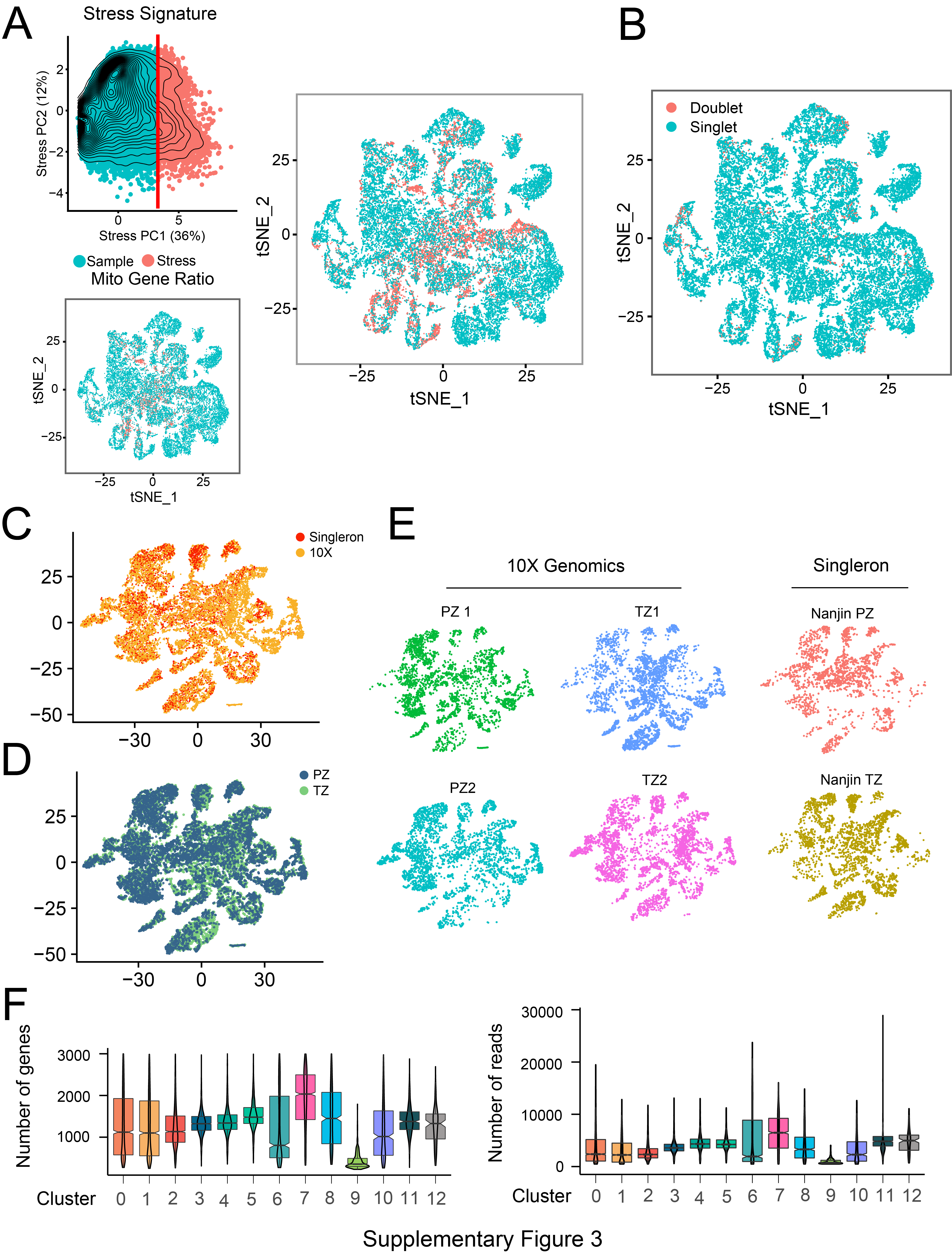

Supplement: Supplementary file 4 — Supporting Information [file CTM2-12-e1084-s014.png]

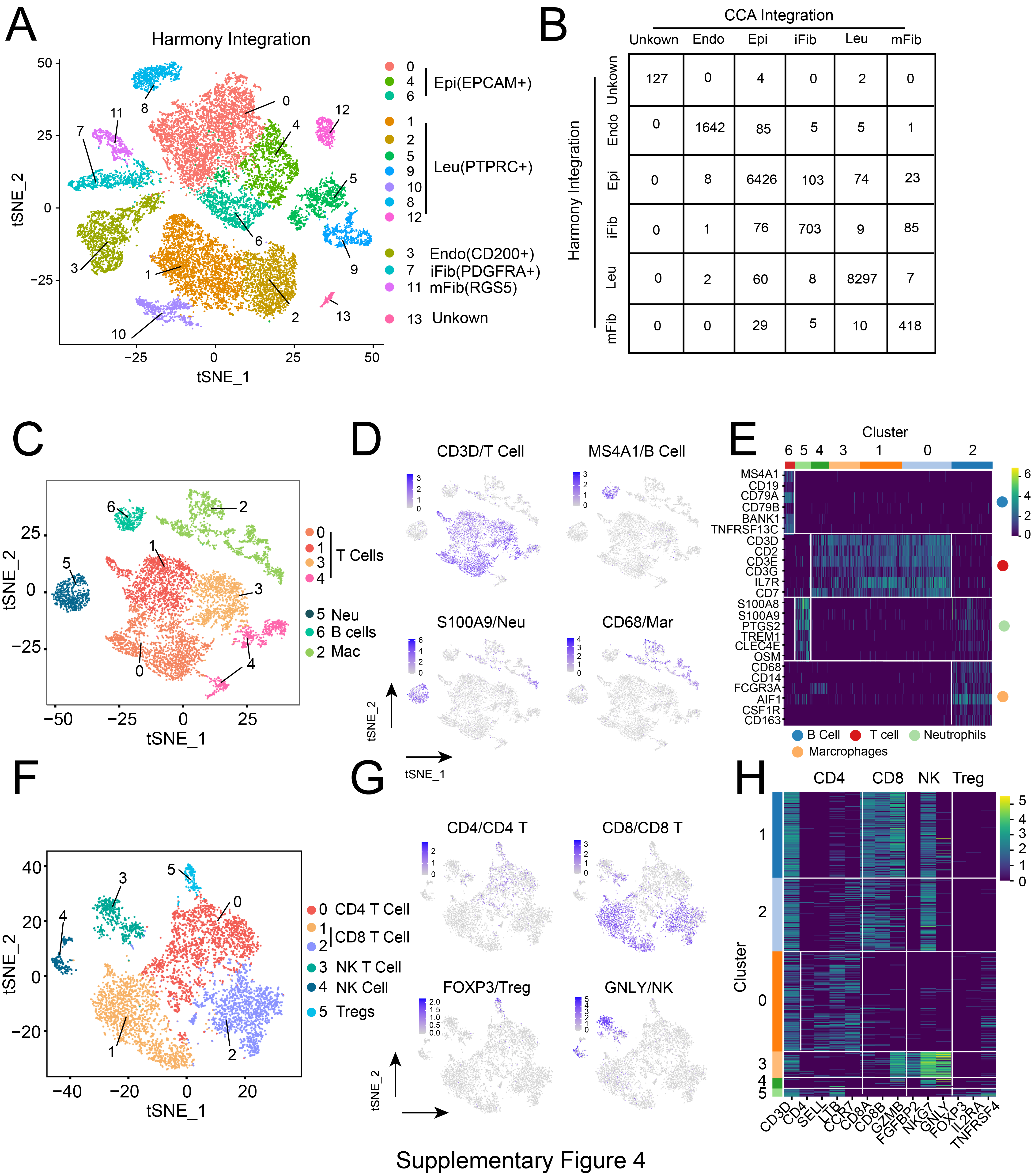

Supplement: Supplementary file 5 — Supporting Information [file CTM2-12-e1084-s011.png]

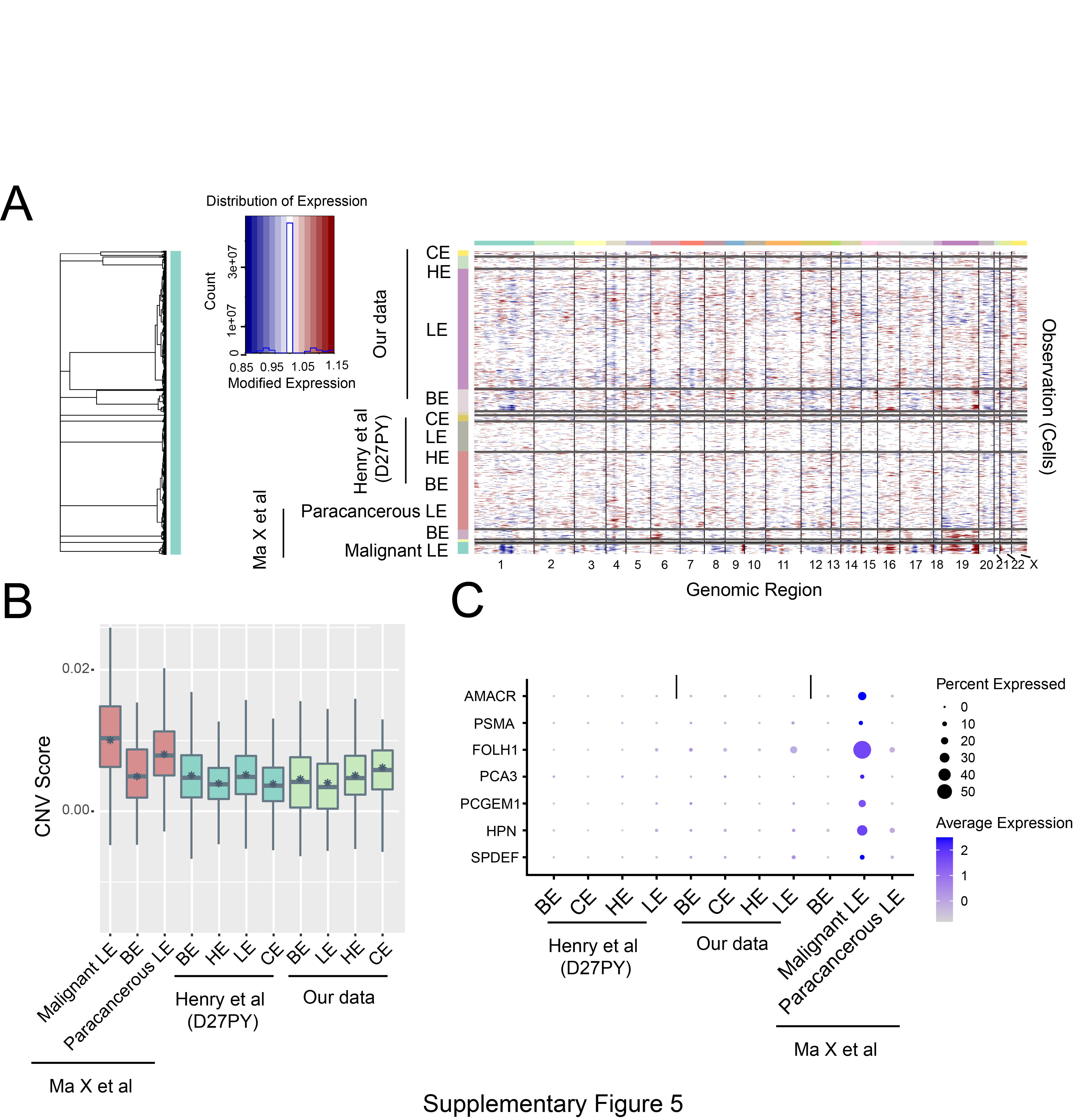

Supplement: Supplementary file 6 — Supporting Information [file CTM2-12-e1084-s012.png]

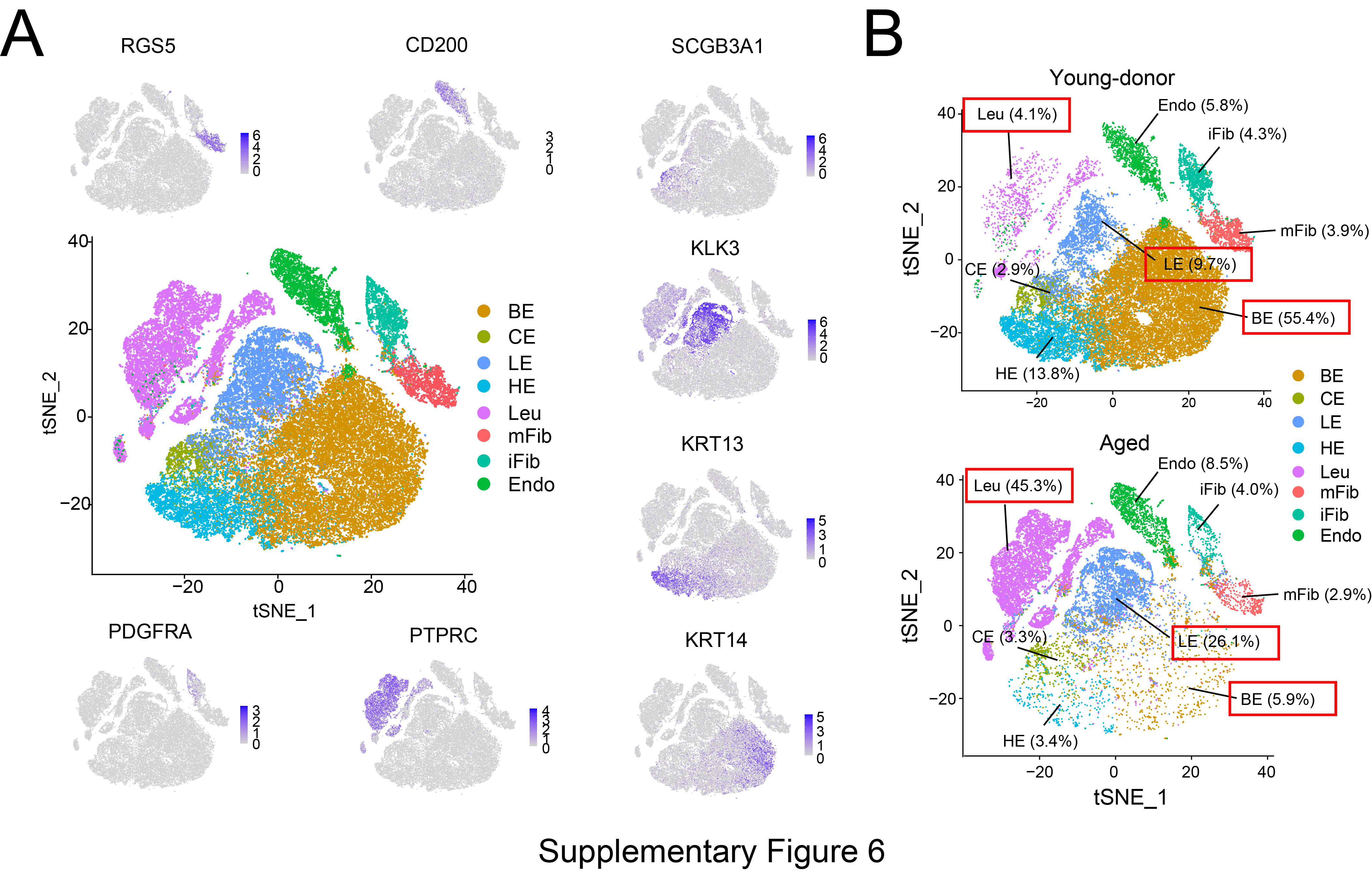

Supplement: Supplementary file 7 — Supporting Information [file CTM2-12-e1084-s010.png]

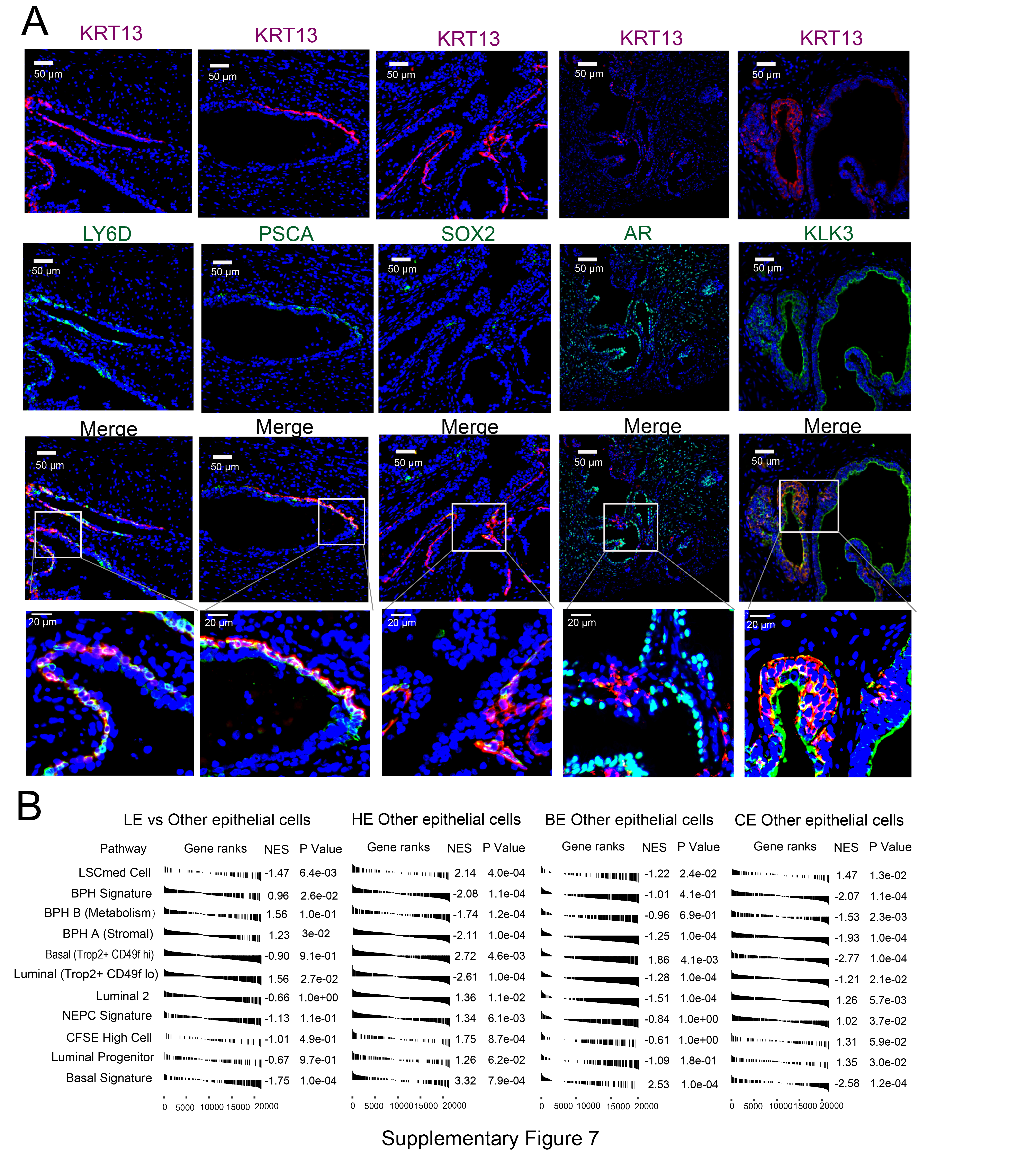

Supplement: Supplementary file 8 — Supporting Information [file CTM2-12-e1084-s001.png]

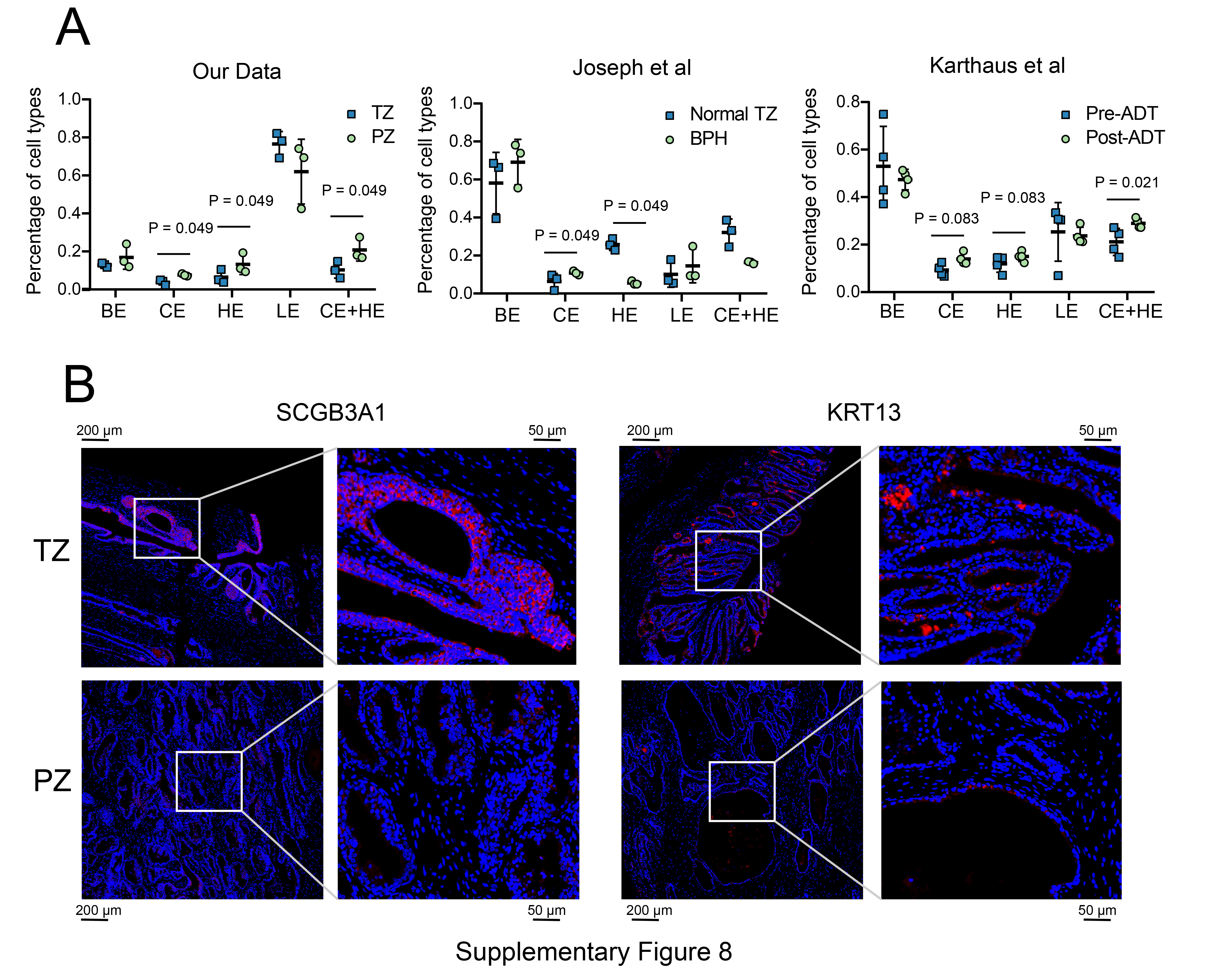

Supplement: Supplementary file 9 — Supporting Information [file CTM2-12-e1084-s004.png]

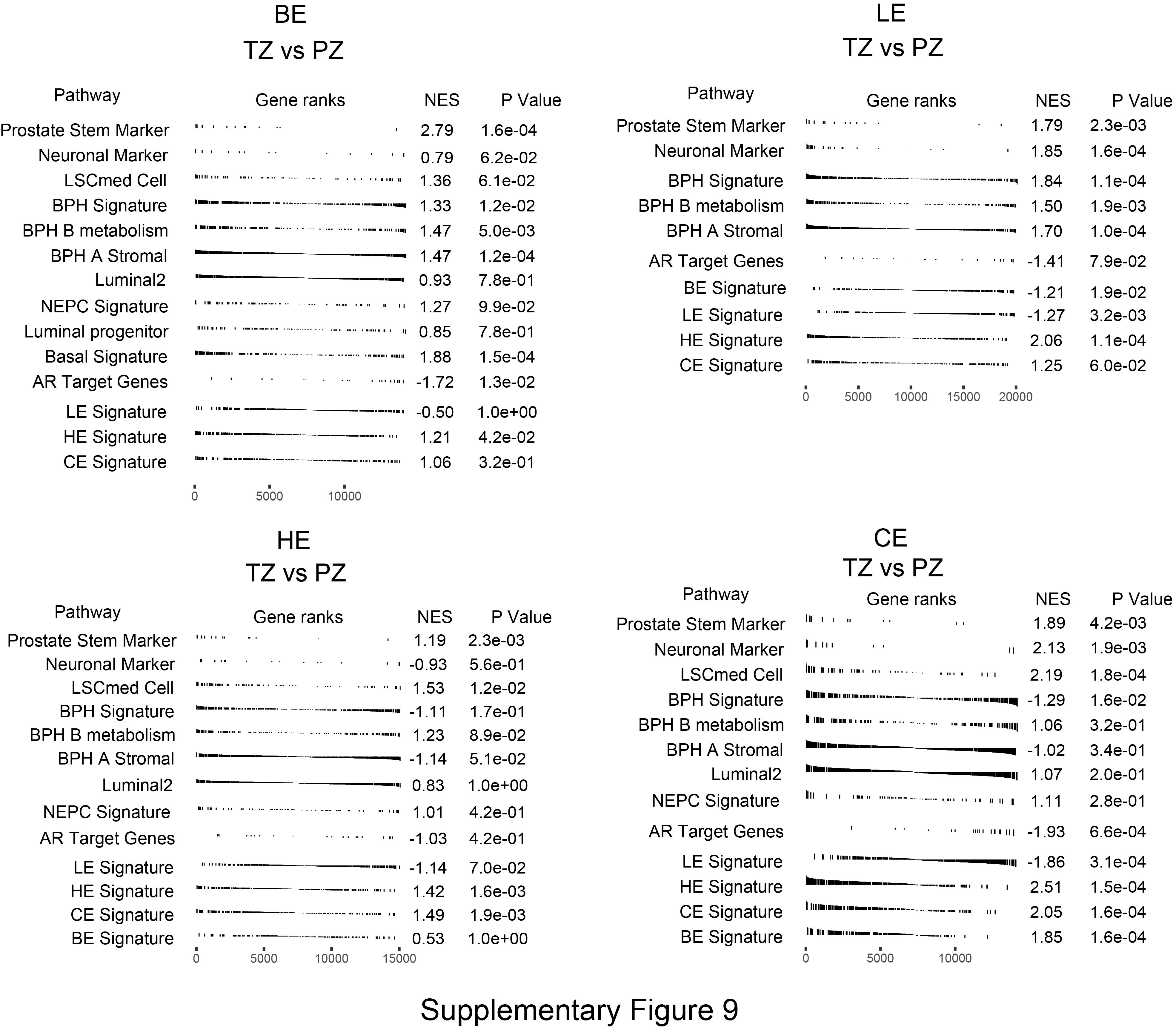

Supplement: Supplementary file 10 — Supporting Information [file CTM2-12-e1084-s005.png]

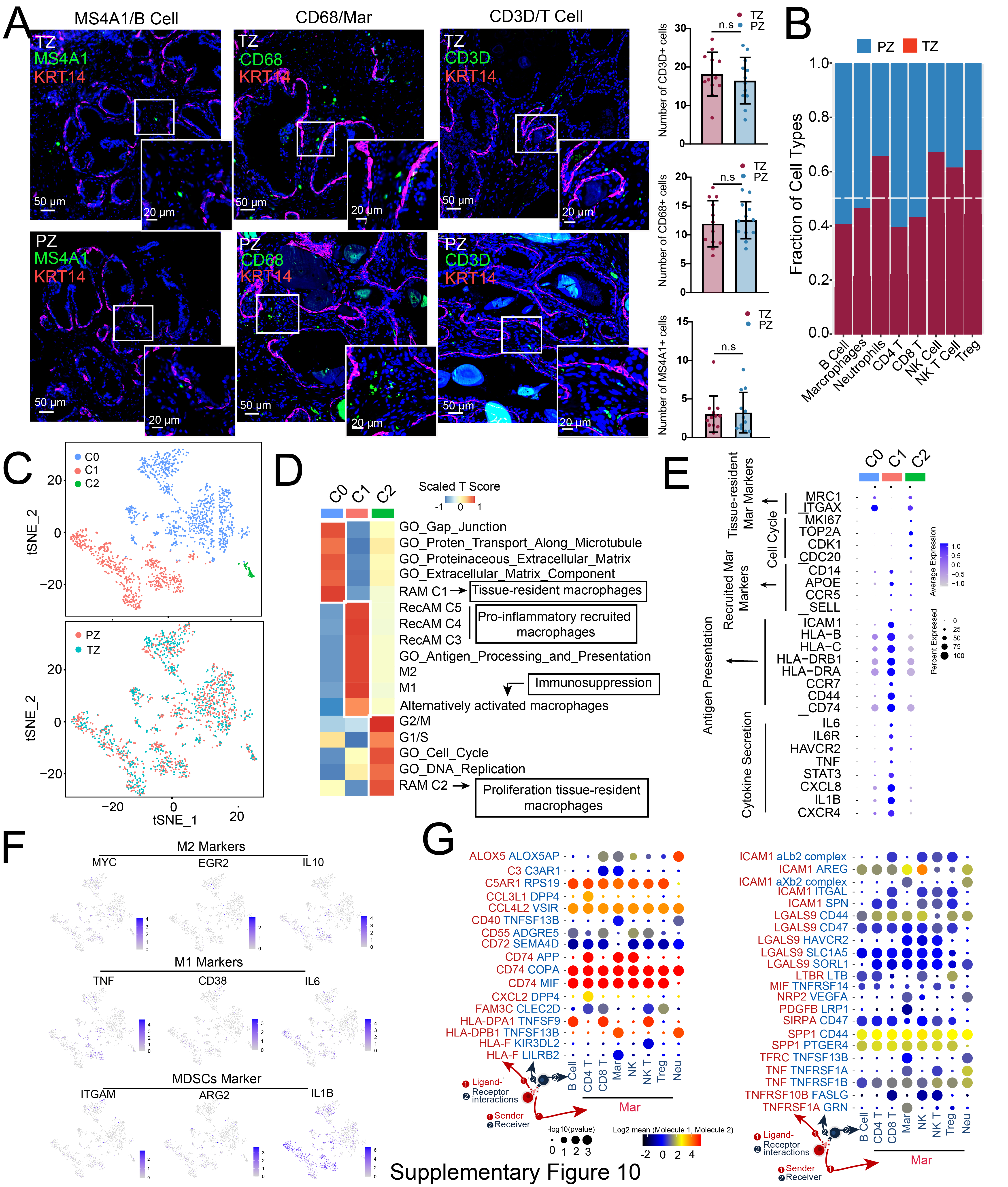

Supplement: Supplementary file 11 — Supporting Information [file CTM2-12-e1084-s006.png]

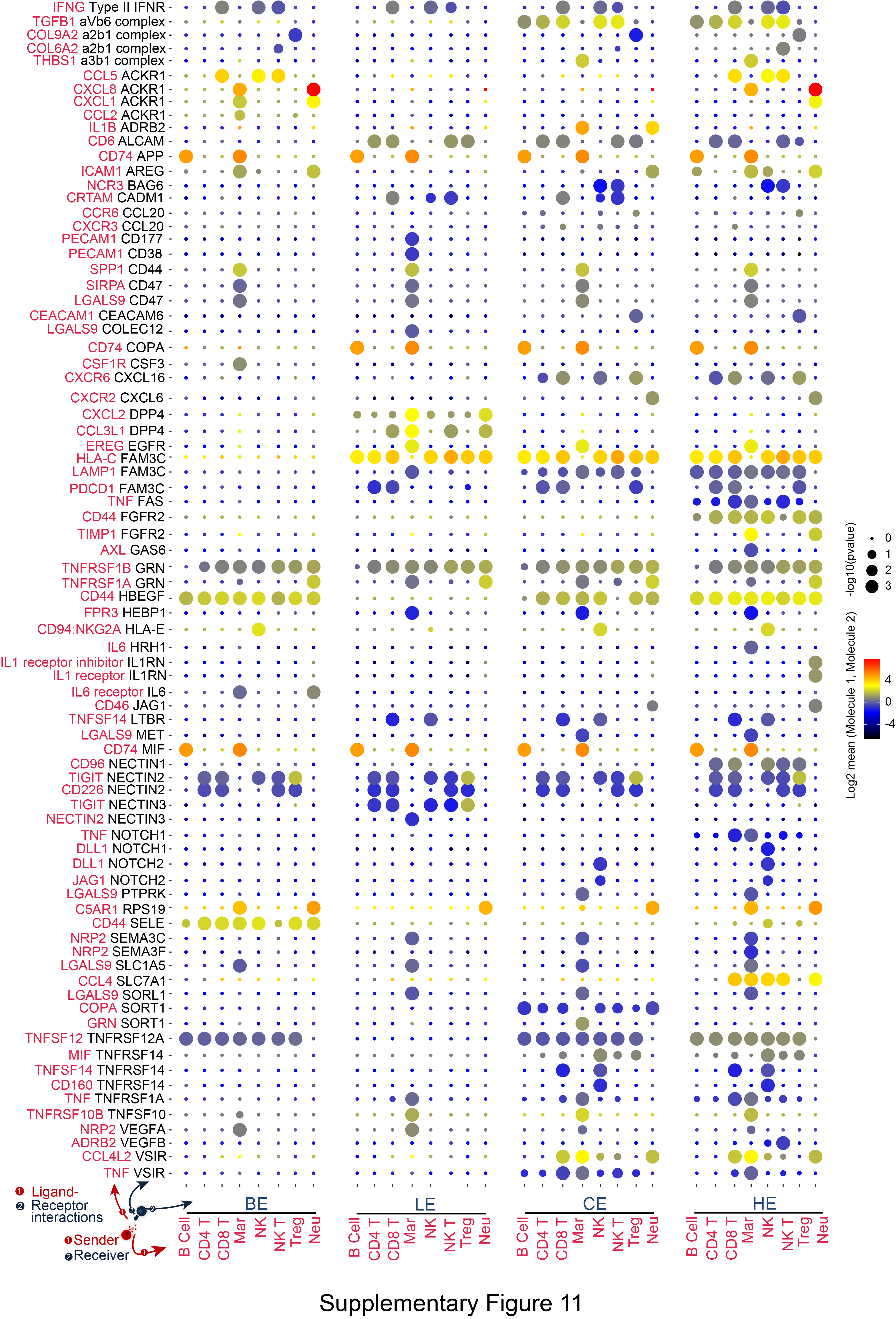

Supplement: Supplementary file 12 — Supporting Information [file CTM2-12-e1084-s003.png]

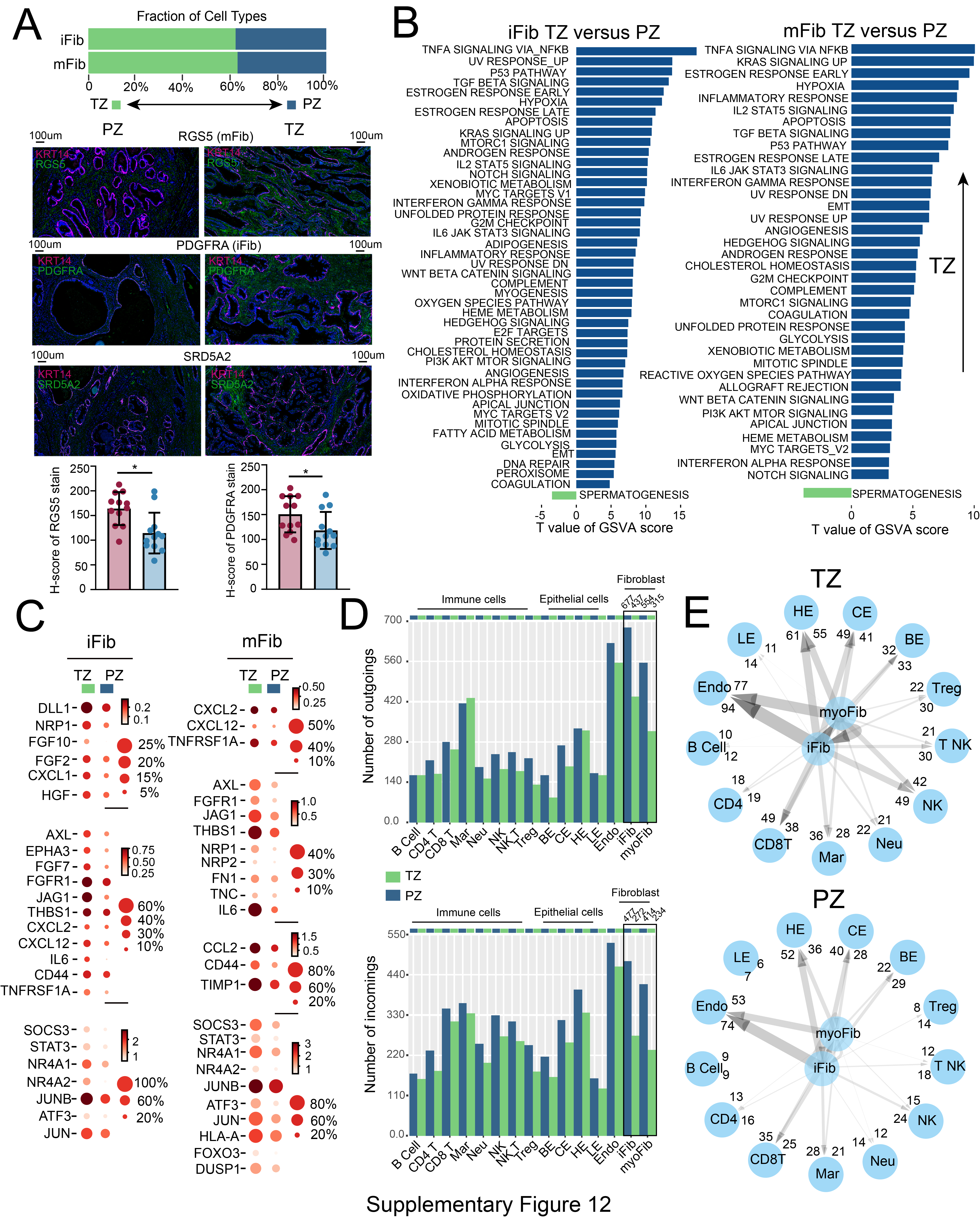

Supplement: Supplementary file 13 — Supporting Information [file CTM2-12-e1084-s008.png]
